# Systematic Review of Climate Change Impacts on Ethiopia's Food Systems: Risk and Vulnerability Assessment

**DOI:** 10.1002/pei3.70207

**Published:** 2026-09-30

**Authors:** Sintayehu Eshetu Abebaw, Tadla Guadie Feleke

**Affiliations:** ^1^ Department of Forestry, College of Agriculture and Natural Resources Mekdela Amba University Tulu Awuliya, Gimba Ethiopia; ^2^ Department of Natural Resources Management, College of Agriculture and Natural Resources Mekdela Amba University Tulu Awuliya, Gimba Ethiopia

**Keywords:** adaptation strategies, agricultural vulnerability, climate change impacts, climate risk assessment, Ethiopia, food insecurity

## Abstract

Climate change is a major driver of food insecurity in Ethiopia, affecting agricultural productivity, water availability, and rural livelihoods. The country's reliance on rain‐fed agriculture increases its vulnerability to climate‐related hazards, underscoring the need to synthesize existing evidence on climate risks and food system vulnerability. This systematic review synthesized scientific evidence on the impacts of climate change on Ethiopia's food systems, identified major climate‐related risks and vulnerability factors, and assessed adaptation strategies and their policy implications. Following PRISMA guidelines, peer‐reviewed studies were systematically identified from major scientific databases using predefined search terms. After applying inclusion and exclusion criteria, 232 eligible studies were included and synthesized thematically to examine climate risks, vulnerability determinants, food system impacts, and adaptation measures. Evidence from 232 studies indicates that climate variability and climate change are major drivers of food insecurity in Ethiopia. Drought, rising temperatures, and changing rainfall patterns were the principal climate risks affecting agricultural production and food availability. Recurrent droughts accounted for approximately 60% of crop yield losses, food insecurity increased by an estimated 2.3% annually, and a 1°C rise in temperature was associated with a 10% reduction in major staple crop production. Rural households were disproportionately affected, experiencing 15% greater deterioration in food security than urban households. The reviewed studies consistently identified climate‐resilient agricultural practices, irrigation expansion, and region‐specific adaptation measures as key strategies for reducing climate‐induced food insecurity. Climate change poses substantial risks to Ethiopia's food systems through interacting environmental and socio‐economic pathways. Strengthening climate‐resilient agriculture, improving water resource management, expanding social protection, and promoting locally appropriate adaptation strategies are essential to enhance food security. These findings provide evidence to inform policy, practice, and future research while highlighting the need to address regional knowledge gaps and evaluate the long‐term effectiveness of adaptation interventions.

## 1 Introduction

### 1.1 Background

Food insecurity continues to be a critical challenge in Ethiopia, driven by a complex interaction of climatic variability, environmental degradation, and socio‐economic difficulties. In 2023, more than 20 million Ethiopians experienced food insecurity as a result of recurring droughts, floods, conflict, and other climate‐related disasters (FAO 2023a, 2023b, 2023c). The nation's heavy reliance on rain‐fed agriculture, which employs approximately 85% of the labor force and contributes about one‐third of the national gross domestic product, makes Ethiopia particularly vulnerable to climate‐related risks (World Bank 2022a, 2022b, 2022c). Annual fluctuations in rainfall, with deviations of approximately ±20% in major agricultural regions, further increase the likelihood of crop failures, thereby threatening the food security and livelihoods of millions of households (IPCC 2021).

Ethiopia has experienced a consistent rise in average temperatures, with an increase of approximately 1.3°C over the past five decades, accompanied by increasingly variable and unpredictable rainfall patterns (National Meteorology Agency of Ethiopia 2020). These climatic changes have adversely affected agricultural productivity, contributing to reductions in cereal yields of up to 15% during severe drought years (CSA 2019). Smallholder farmers, who constitute nearly 90% of the country's agricultural producers, are particularly vulnerable because of their dependence on rain‐fed farming, limited access to irrigation, financial constraints, and restricted adaptive capacity (IFPRI 2021).

Agriculture remains the backbone of Ethiopia's economy, with nearly 80% of the population depending directly or indirectly on smallholder agriculture for food and income (FAO 2021a, 2021b, 2021c, 2021d). However, the sector's dependence on rain‐fed production systems makes it highly susceptible to recurrent droughts, floods, erratic rainfall, heat stress, and land degradation. These climatic hazards reduce agricultural productivity, disrupt rural livelihoods, and impede national progress toward achieving Sustainable Development Goal 2 (Zero Hunger) and other climate‐resilient development objectives.

Climate change is further increasing both the frequency and intensity of these hazards, creating cumulative impacts across the entire food system. Ethiopia's vulnerability is compounded by low adaptive capacity, widespread poverty, dependence on climate‐sensitive natural resources, environmental degradation, and fragile ecosystems (Teku and Abebaw 2024). Climate‐related shocks not only reduce crop and livestock productivity but also affect food processing, transportation, market access, storage systems, household purchasing power, and food prices, thereby influencing all four dimensions of food security: availability, access, utilization, and stability (Shiferaw et al. 2014; IPCC 2022a, 2022b).

Recent global assessments show that climate change is disrupting entire food systems by affecting agricultural production, supply chains, food environments, dietary quality, and socioeconomic stability. The IPCC Sixth Assessment Report (AR6) concludes with very high confidence that Africa is among the regions most vulnerable to climate‐related food insecurity due to high exposure, strong dependence on climate‐sensitive livelihoods, and limited adaptive capacity (IPCC 2022a, 2022b). Similarly, the State of Food Security and Nutrition in the World (SOFI) identifies climate extremes as a major driver of persistent food insecurity, particularly in predominantly rain‐fed agricultural systems such as Ethiopia (FAO, International Fund for Agricultural Development (IFAD), et al. 2023). Although these assessments consistently identify climate change as a key driver of food insecurity, evidence from Ethiopia indicates that climate risks vary across agro‐ecological zones, with drought predominating in moisture‐deficit areas, whereas rainfall variability, flooding, and temperature extremes increasingly constrain humid and highland production systems. This highlights that climate risks are interacting and location‐specific rather than driven by a single climatic hazard (IPCC 2022a, 2022b; FAO, International Fund for Agricultural Development (IFAD), et al. 2023).

Understanding how climate risks intersect with food insecurity in Ethiopia is therefore essential for addressing this persistent national challenge. Existing studies consistently recommend integrating climate risk assessments into national food security planning and climate adaptation policies to strengthen resilience (UNDP 2022). Despite the growing number of empirical studies conducted across Ethiopia, the evidence remains fragmented, geographically dispersed, and methodologically heterogeneous. Most studies focus on specific climate hazards, crops, localized drought impacts, household food insecurity, or adaptation practices, which limits a comprehensive understanding of climate change impacts on Ethiopia's food systems. Although narrative reviews have examined climate change adaptation, agricultural resilience, food security, and sustainable agriculture, they largely emphasize specific sectors or interventions rather than systematically synthesizing climate‐related risks across the entire food system. Similarly, international systematic reviews provide limited country‐specific evidence relevant to Ethiopia's biophysical, socioeconomic, and institutional context, leaving policymakers without an integrated evidence base on major climate hazards, vulnerable populations, affected food system components, adaptation responses, and knowledge gaps.

Previous studies consistently show that climate change negatively affects all dimensions of food security, but they differ in explaining the underlying mechanisms. Although some attribute food insecurity mainly to declining agricultural production, others emphasize market disruptions, household income losses, rising food prices, and reduced livelihood diversification, highlighting the need for integrated food‐system assessments that capture both biophysical and socioeconomic processes. This review addresses these gaps by systematically synthesizing available evidence on climate change impacts across Ethiopia's food systems using a structured and transparent methodology. Unlike previous reviews, it simultaneously examines climate hazards, exposure pathways, vulnerability factors, adaptation strategies, and food system outcomes, covering food availability, access, utilization, stability, livelihoods, and socioeconomic vulnerability to provide a more comprehensive assessment of climate‐related risks.

Furthermore, the review compares methodological approaches across studies and shows an increasing use of household surveys, spatial analyses, climate records, vulnerability indices, and geospatial techniques. However, inconsistencies in study design, food insecurity indicators, and vulnerability assessment frameworks continue to limit comparability across studies and remain important priorities for future research. Given these challenges, conducting systematic climate risk assessments is vital for understanding vulnerabilities within Ethiopia's food systems and for designing effective adaptation and mitigation strategies. Such assessments evaluate the interaction between climate hazards, exposure, sensitivity, and adaptive capacity to estimate potential impacts on food security (IPCC 2022a, 2022b).

For Ethiopia, where recurrent droughts, increasing rainfall variability, rapid population growth, and dependence on climate‐sensitive agriculture intersect, synthesizing scientific evidence is crucial for informing policy, climate‐resilient agricultural planning, disaster risk reduction, and investment prioritization. This systematic review identifies consistent patterns, regional variations, methodological limitations, and research priorities that individual studies cannot capture. It provides one of the first comprehensive syntheses of climate change impacts on Ethiopia's food systems from a risk and vulnerability perspective. By integrating evidence across agro‐ecological zones, climatic hazards, agricultural systems, and food security dimensions, the review offers a consolidated evidence base for strengthening climate‐resilient food systems, addressing knowledge gaps, and supporting Ethiopia's commitments to the Sustainable Development Goals, the Paris Agreement, and national climate resilience strategies.

### 1.2 Theoretical Framework

The theoretical framework for assessing the intersection between climate change and food security in Ethiopia is grounded in internationally recognized concepts of climate risk, vulnerability, resilience, and food systems. This framework integrates established theories of climate change vulnerability with the four dimensions of food security to provide a comprehensive basis for synthesizing evidence from the reviewed literature. Food insecurity is conceptualized as an outcome of interactions among climate hazards, exposure, sensitivity, adaptive capacity, and vulnerability, rather than as independent climate hazards or food security indicators. This integrated approach aligns with the Intergovernmental Panel on Climate Change (IPCC) risk framework and the Food and Agriculture Organization (FAO) food security framework (IPCC 2022a, 2022b; FAO 2008).

#### 1.2.1 Climate Risk Components

Climate risk consists of three interrelated components: exposure, sensitivity, and adaptive capacity, which together determine the degree of vulnerability of households, agricultural systems, and food systems to climate change (IPCC 2014, 2022a, 2022b). Exposure refers to the extent to which food systems are subjected to climate‐related hazards such as droughts, floods, heat waves, erratic rainfall, and increasing temperatures. Sensitivity describes the degree to which these hazards affect agricultural production and food security, depending on factors including dependence on rain‐fed agriculture, soil degradation, ecosystem conditions, poverty, and livelihood characteristics. Adaptive capacity refers to the ability of individuals, households, communities, and institutions to anticipate, respond to, and recover from climate‐related disturbances through access to technology, financial resources, infrastructure, education, governance, and institutional support (Adger et al. 2005).

In the IPCC Sixth Assessment Report, climate risk is understood as the result of interactions among climate hazards, exposure, vulnerability, and response capacity. Consequently, vulnerability is not viewed as an isolated characteristic but as a dynamic condition shaped by the combined effects of exposure, sensitivity, and adaptive capacity. This perspective provides an appropriate conceptual basis for understanding why similar climatic events produce different food security outcomes across regions, livelihood groups, and agroecological zones within Ethiopia (IPCC 2022a, 2022b).

#### 1.2.2 Food Security Dimensions

Food security is traditionally defined through four interconnected dimensions: food availability, food access, food utilization, and food stability (FAO 2008). Climate change influences each of these dimensions through multiple pathways.

1. *Availability*: Climate‐induced reductions in crop and livestock productivity decrease food production and limit the availability of food at household and national levels (Schmidhuber and Tubiello 2007).
2. *Accessibility*: Climatic shocks reduce household income, damage infrastructure, disrupt markets, and increase food prices, thereby limiting both physical and economic access to food (Barrett 2010).
3. *Utilization*: Climate variability affects dietary diversity, food safety, water quality, sanitation, and the prevalence of climate‐sensitive diseases, thereby influencing the nutritional utilization of available food (Lobell et al. 2008).
4. *Stability*: Recurrent droughts, floods, and seasonal climate variability undermine the long‐term stability of food production, food supply chains, household livelihoods, and market systems, resulting in persistent food insecurity (Ericksen 2008).

These four dimensions are highly interconnected. Climate‐related disturbances affecting agricultural production often trigger cascading effects throughout the food system, influencing market availability, purchasing power, nutritional outcomes, and the long‐term resilience of vulnerable households. Therefore, understanding climate impacts requires consideration of the entire food system rather than agricultural production alone (IPCC 2022a, 2022b; FAO et al. 2023).

#### 1.2.3 Integrating Climate Risk, Vulnerability, and Food Security

The relationship between climate risk components and food security dimensions is complex, multidimensional, and context specific. Previous studies conducted in Ethiopia demonstrate considerable regional variation in climate exposure, livelihood sensitivity, and adaptive capacity. Drought‐prone areas frequently experience substantial reductions in agricultural production and food availability, whereas households with limited financial resources, weak institutional support, and low access to climate‐resilient technologies experience greater food insecurity (Deressa et al. 2009a, 2009b, 2009c; Conway and Schipper 2011). Adaptive capacity plays a particularly important role in moderating climate impacts on food security. Evidence indicates that interventions such as irrigation development, climate‐smart agricultural practices, improved seed varieties, livelihood diversification, early warning systems, and strengthened extension services can reduce household vulnerability and improve resilience to climate variability (Gebrehiwot and van der Veen 2013a, 2013b, 2013c).

Accordingly, this theoretical framework serves as the organizing structure for the present systematic review. Evidence synthesized from the included studies is interpreted according to five interrelated analytical components: (i) exposure to climate hazards, (ii) sensitivity of agricultural and food systems, (iii) adaptive capacity of households and institutions, (iv) resulting vulnerability, and (v) impacts on the four dimensions of food security. This structure enables consistent interpretation of evidence across studies despite differences in research design, geographical coverage, and methodological approaches.

#### 1.2.4 Theoretical Implications

This framework draws upon both vulnerability theory and resilience theory to explain how interactions among climate hazards, socioeconomic conditions, and institutional capacities shape food security outcomes. It emphasizes that strengthening adaptive capacity while reducing exposure and sensitivity is essential for enhancing resilience within Ethiopia's food systems and achieving sustainable development objectives. The framework provides the conceptual basis for synthesizing and interpreting findings in this review. The Results and Discussion sections are structured around exposure, sensitivity, adaptive capacity, vulnerability, and the four dimensions of food security, with each finding linked to these elements. This ensures a transparent, theory‐driven interpretation of evidence, enhances consistency between objectives, synthesis, and conclusions, and supports the identification of knowledge gaps and policy implications.

## 2 Methodology

This systematic review was conducted to synthesize the available scientific evidence on the impacts of climate change on Ethiopia's food systems using a structured, transparent, and reproducible review process. The review was designed and reported following the Preferred Reporting Items for Systematic Reviews and Meta‐Analyses (PRISMA 2020) Statement (Page et al. 2021). Because of the considerable heterogeneity among the included studies in terms of study design, outcome variables, analytical methods, geographical coverage, and reported indicators, the findings were synthesized using the Synthesis Without Meta‐analysis (SWiM) reporting guideline rather than a quantitative meta‐analysis (Campbell et al. 2020).

### 2.1 Literature Search Strategy

A comprehensive literature search was conducted using major scientific databases, including Google Scholar, Scopus, and PubMed to identify relevant studies on climate change and food systems in Ethiopia. The search covered studies published between 2000 and 2026. The search strategy combined keywords and Boolean operators to maximize retrieval of relevant literature. The principal search terms included combinations of “climate change,” “climate variability,” “food systems,” “food security,” “agriculture,” “vulnerability,” “risk assessment,” “adaptation,” and “Ethiopia.” Boolean operators such as AND and OR were used to combine search terms according to the requirements of each database.

An example of the search string was:

(“Climate change” OR “Climate variability” OR drought OR flood) AND (“Food security” OR “Food systems” OR agriculture) AND (Ethiopia). Reference lists of eligible articles were also examined to identify additional relevant studies that were not captured during the database searches. The final database search was completed before manuscript preparation. The complete study selection process is presented in the PRISMA 2020 flow diagram (Figure 1).

**FIGURE 1:**
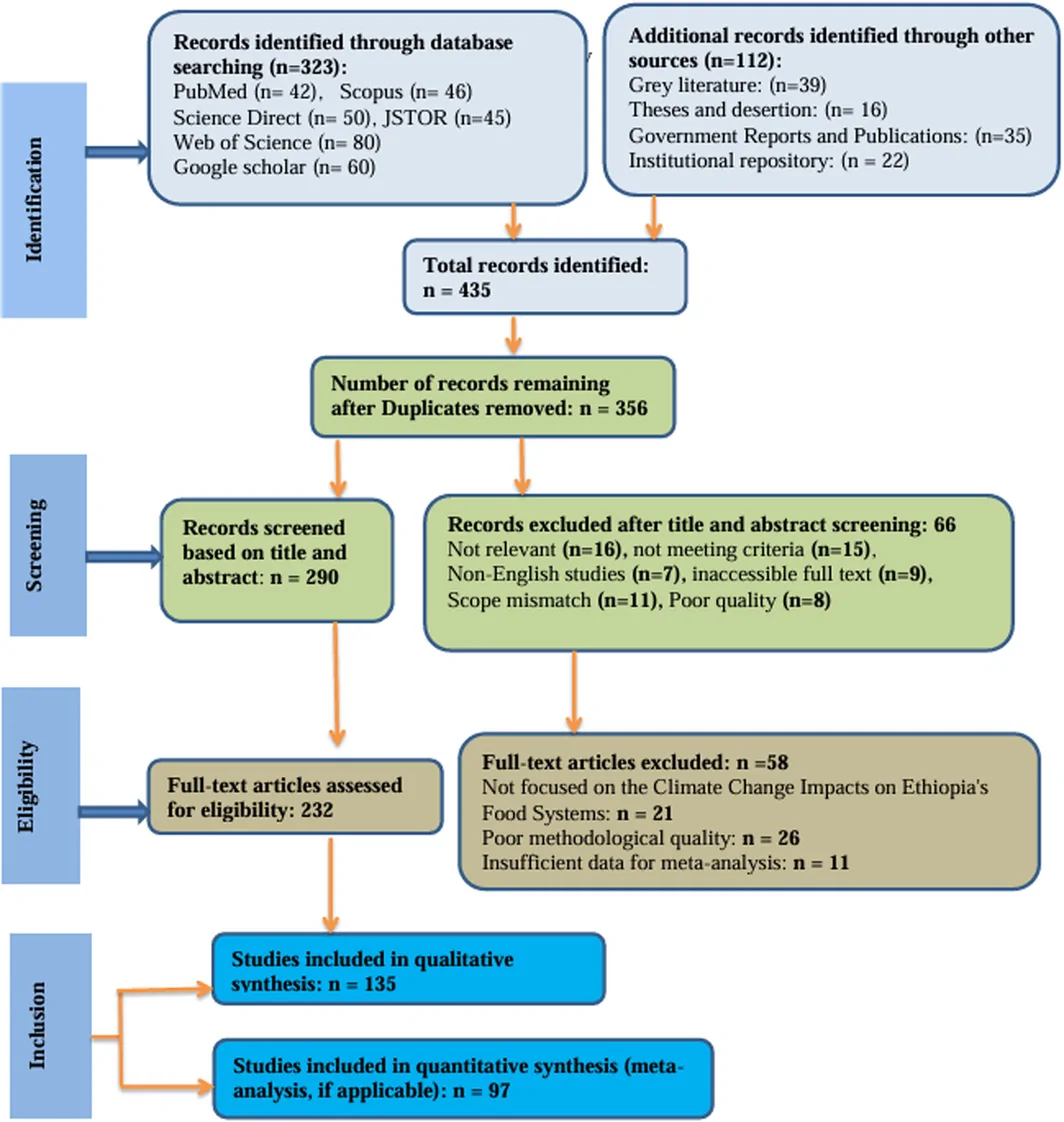
PRISMA 2020 flow diagram illustrating the study selection process.

### 2.2 Eligibility Criteria

Studies were selected according to predefined inclusion and exclusion criteria established before the screening process to ensure transparency, consistency, and reproducibility throughout the systematic review (Page et al. 2021; Campbell et al. 2020).

Studies were included if they:

1. were published in peer‐reviewed journals;
2. investigated climate change, climate variability, or climate‐related hazards affecting food systems or food security;
3. focused on Ethiopia;
4. reported empirical findings using qualitative, quantitative, or mixed‐method research designs; and
5. were published in English between 2000 and 2026.

Studies were excluded if they:

1. were conducted outside Ethiopia;
2. were conference abstracts, editorials, commentaries, theses, book chapters, or review articles;
3. lacked sufficient methodological information or empirical findings to address the review objectives; or
4. were unavailable in full text.

These eligibility criteria were developed to identify studies directly relevant to the review objectives while minimizing the inclusion of studies with insufficient methodological rigor or inadequate reporting. The study selection process followed the recommendations of the PRISMA 2020 statement, and the synthesis of findings was conducted in accordance with the Synthesis Without Meta‐analysis (SWiM) reporting guideline (Page et al. 2021; Campbell et al. 2020).

### 2.3 Study Screening and Selection

All retrieved records were exported and screened systematically. Duplicate records were identified and removed before screening. The remaining studies underwent title screening followed by abstract screening to determine their relevance to the review objectives. Full‐text articles meeting the eligibility criteria were then assessed for final inclusion. Studies that satisfied all inclusion criteria were included in the final synthesis. The complete screening and selection procedure is summarized using the PRISMA 2020 flow diagram (Figure 1).

### 2.4 Data Extraction

Data extraction was performed using a standardized data extraction template developed for this review. The extracted information included author(s), publication year, study location, study design, objectives, climate‐related hazards investigated, food system components examined, and indicators of food security, vulnerability factors, adaptation strategies, principal findings, and major conclusions. Where available, descriptive quantitative information such as percentages, frequencies, or reported changes in agricultural production and food security indicators was also recorded. To improve consistency and minimize extraction errors, the extracted information was checked against the original publications before inclusion in the final synthesis.

### 2.5 Risk Assessment Tools and Frameworks

The present study did not apply a primary climate risk assessment model because it is a systematic review that synthesizes evidence from previously published empirical studies. Instead, the review examined the risk assessment approaches and conceptual frameworks adopted by the included studies to evaluate the impacts of climate change on Ethiopia's food systems. Many of the included studies assessed climate‐related risks using the Intergovernmental Panel on Climate Change (IPCC) risk framework, which conceptualizes climate risk as the interaction among climate hazards, exposure, vulnerability, and adaptive capacity (IPCC 2022a, 2022b). Within this framework, vulnerability is determined by the degree to which households, communities, or agricultural systems are exposed and sensitive to climate hazards and their capacity to adapt to changing climatic conditions.

The reviewed studies employed a range of assessment approaches, including analyses of historical climate data (e.g., rainfall variability and temperature trends), household surveys, socioeconomic vulnerability assessments, agricultural productivity indicators, food security measurements, and adaptation assessments. These approaches were used to examine the effects of climate‐related hazards such as droughts, floods, erratic rainfall, increasing temperatures, and extreme weather events on agricultural production, food availability, food access, food utilization, and livelihood resilience.

During evidence synthesis, information on the climate hazards investigated, vulnerability indicators, exposure factors, adaptive capacity measures, and reported food system impacts was systematically extracted and organized into thematic categories. This approach enabled comparison of findings across studies and facilitated the identification of common climate risks, vulnerable populations, adaptation strategies, and research gaps affecting Ethiopia's food systems.

### 2.6 Quality Assessment

The methodological quality of the included studies was evaluated during the full‐text review to ensure that only relevant and reliable evidence was included in the synthesis. Each study was assessed based on predefined methodological criteria, including clarity of the study objectives, appropriateness of the study design, description of the study area and population, adequacy of data collection methods, transparency of data analysis, consistency between the reported results and conclusions, and relevance to the objectives of this systematic review.

Studies with insufficient methodological information, unclear data sources, unsupported conclusions, or inadequate reporting were carefully examined and excluded when they did not provide sufficient evidence to address the review objectives. Only studies that met the predefined eligibility criteria and demonstrated adequate methodological quality were included in the final synthesis. The quality assessment was conducted to improve the credibility and transparency of the review findings and to minimize the inclusion of studies with substantial methodological limitations. The assessment focused on evaluating the overall reliability and relevance of the available evidence rather than assigning numerical quality scores or excluding studies solely on the basis of reporting characteristics (Page et al. 2021; Higgins et al. 2022).

Each study was assessed using the following questions:

1. Was the study objective clearly stated?
2. Were the climate data sources or climate models adequately described?
3. Were the analytical methods or modeling procedures clearly explained and scientifically appropriate?
4. Were model validation procedures or uncertainty analyses reported where applicable?
5. Were the results presented clearly and supported by sufficient evidence?
6. Were the conclusions consistent with the reported findings?

Each criterion received a score of Yes (1) or No (0), resulting in a maximum possible score of six.

The inclusion decision was determined using predefined thresholds:

**Table:** 

Quality score	Decision
5–6 Yes	High‐quality study; included
3–4 Yes	Moderate‐quality study; included after careful evaluation
0–2 Yes	Low‐quality study; excluded

Accordingly, studies receiving only two or fewer “Yes” responses were excluded, whereas studies scoring three or more “Yes” responses were retained for evidence synthesis. This procedure ensured that only studies demonstrating acceptable methodological quality contributed to the final review while reducing the risk of bias.

### 2.7 Data Synthesis

Because the included studies differed substantially with respect to research design, study populations, geographic locations, climate variables, food system indicators, outcome measures, and analytical approaches, statistical pooling of results was not appropriate. Consequently, no meta‐analysis was conducted. Instead, the findings of the selected studies were synthesized using tables, frequencies, charts, and graphs. Data extracted from the included articles were systematically organized according to study characteristics, climate change impacts, food system components, climate‐related hazards, vulnerability factors, and adaptation strategies. Similar findings across studies were grouped into common themes and summarized to identify consistent patterns, major trends, and research gaps regarding the impacts of climate change on Ethiopia's food systems. Where quantitative information, including percentages, frequencies, or reported changes in agricultural production and food security indicators, was available, these findings were summarized descriptively without statistical pooling. The narrative synthesis followed the principles of the SWiM (Synthesis Without Meta‐analysis) reporting guideline by transparently describing how studies were grouped, how evidence was summarized, and how conclusions were derived from the included literature (Campbell et al. 2020). This approach enabled a comprehensive assessment of climate‐related risks, vulnerabilities, adaptation strategies, and their implications for Ethiopia's food systems while acknowledging the methodological diversity of the available evidence.

## 3 Result and Findings

### 3.1 History and Extent of Food Insecurity in Ethiopia

Ethiopia has a long history of food insecurity, driven by recurrent droughts, rapid population growth, environmental degradation, conflict, weak market systems, and persistent poverty. Severe famines have repeatedly disrupted food production and rural livelihoods, with major crises occurring during the 1970s, 1980s, and early 2000s. Between 1958 and 1977, more than 25 million people were affected by drought and famine, resulting in an estimated 3–5 million deaths (Berhanu 2001). The 1983–1985 famine remains one of the most devastating humanitarian disasters in the country's history, affecting approximately 8 million people and causing nearly 1 million deaths (De Waal 1991; Berhanu 2001). Likewise, the 2002–2003 drought affected nearly 13 million people, with approximately 6 million requiring immediate emergency food assistance (Berhanu 2001).

Despite sustained economic growth over the past two decades, food insecurity remains widespread. According to FAO (2023), approximately 20.6% of Ethiopia's population remained undernourished during 2020–2022, indicating persistent challenges in ensuring adequate food access. Ethiopia's agricultural sector continues to employ nearly 70% of the labor force and contributes approximately one‐third of the national Gross Domestic Product, whereas over 95% of crop production depends on rainfall, making agricultural production highly sensitive to climate variability and extreme weather events (World Bank 2021a, 2021b, 2021c, 2021d; FAO 2023a, 2023b, 2023c). Recent estimates further indicate that more than 20 million Ethiopians required humanitarian food assistance during 2023 owing to the combined effects of drought, conflict, inflation, and displacement (WFP 2023a, 2023b, 2023c; OCHA 2023).

Within the conceptual framework adopted in this review, the historical evolution of food insecurity reflects the interaction between climate exposure, household sensitivity, and adaptive capacity. Ethiopia experiences repeated exposure to droughts, floods, increasing temperatures, and rainfall variability. These climatic hazards interact with high dependence on rain‐fed agriculture, widespread rural poverty, and limited livelihood diversification, thereby increasing household sensitivity to food insecurity. Adaptive capacity has improved through national interventions such as the Productive Safety Net Programme (PSNP), which has supported approximately 8 million chronically food‐insecure people annually by providing predictable transfers and promoting livelihood resilience (MoA 2020; World Bank 2021a, 2021b, 2021c, 2021d). Nevertheless, recurrent climate shocks combined with conflict and rising food prices continue to outpace existing adaptation efforts.

Recent quantitative evidence highlights the increasing severity of climate‐induced food insecurity in Ethiopia. Between late 2020 and early 2023, consecutive droughts across southern and eastern regions led to the failure of up to five rainy seasons, widespread crop loss, death of millions of livestock, and severe declines in household food availability (FAO 2023a, 2023b, 2023c; WFP 2023a, 2023b, 2023c). National assessments further report that over 10 million livestock were lost due to prolonged drought, significantly reducing income, dietary diversity, and resilience among pastoral and agro‐pastoral households (FAO 2023a, 2023b, 2023c).

Climate model projections consistently show that future warming and rainfall variability will further undermine agricultural production, with maize yield declines projected at about 10%–30% under high‐emission scenarios by mid‐ to late‐century, depending on adaptation measures, cultivars, and climate pathways (Adhikari et al. 2015; Asfaw and Bedemo 2022a; Araya et al. 2015; Sultan et al. 2019). These losses pose a direct threat to food availability and access, particularly for rain‐fed smallholder farming systems.

Figure 2, illustrates regional variations in the number of people requiring emergency food assistance in Ethiopia during 2015 and 2016 Adapted from Climate Risk Management in Africa: Learning from Practice, 2007, and subsequent humanitarian food security assessment data (IRI), 2007. The highest numbers of food‐insecure people were recorded in Oromia, followed by Amhara, Somali, and Tigray, indicating that these regions were the most severely affected by recurrent drought, rainfall failure, and climate‐related shocks. Between 2015 and 2016, the number of people requiring relief assistance increased markedly in Oromia, Amhara, and Tigray, reflecting the severe impacts of the 2015–2016 El Niño‐induced drought, which substantially reduced agricultural production, pasture availability, and household food security. In contrast, regions such as Afar experienced a slight decline, whereas Benishangul‐Gumuz, Gambella, Dire Dawa, and Harari recorded relatively low numbers of affected people. Overall, the figure highlights the unequal spatial distribution of food insecurity across Ethiopia and demonstrates that climate variability disproportionately affects regions highly dependent on rain‐fed agriculture and pastoral livelihoods, emphasizing the need for targeted disaster risk management and resilience‐building interventions.

**FIGURE 2:**
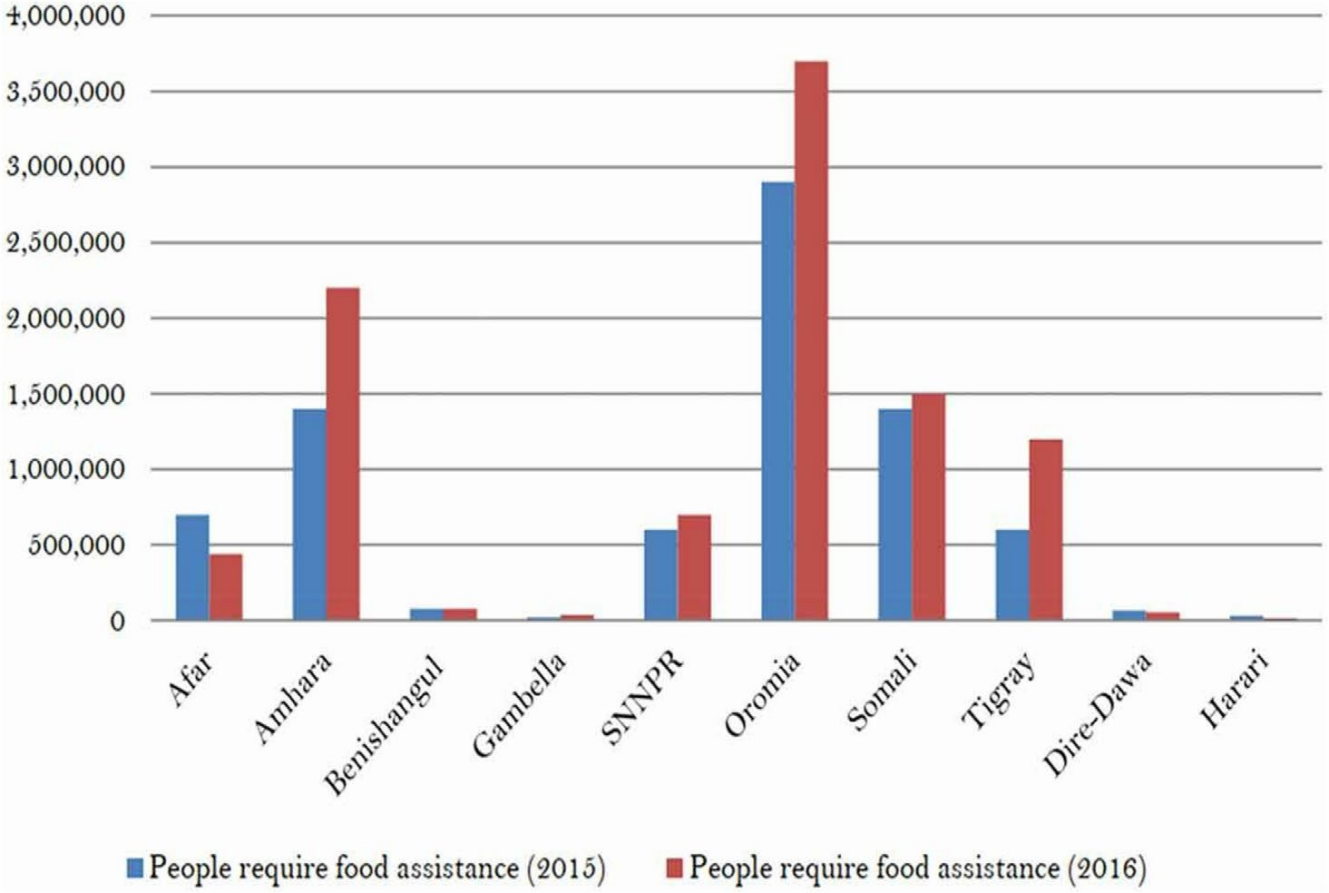
Population requiring relief food assistance by region in Ethiopia, 2015 and 2016.

Similar climate–food insecurity dynamics are evident in other vulnerable regions, indicating that Ethiopia's experience reflects broader global patterns. Across Sub‐Saharan Africa, droughts, rainfall variability, and rising temperatures have reduced productivity and increased food insecurity among smallholder farmers (IPCC 2022a, 2022b). In the Sahel, interacting pressures of climate variability, land degradation, and limited adaptive capacity continue to undermine crop production and resilience (Lalou et al. 2019). In South Asia, temperature extremes, erratic monsoon rainfall, and water scarcity are similarly intensifying agricultural vulnerability and threatening food security among resource‐poor households (Mirzabaev et al. 2023).

These comparisons show that, despite Ethiopia's unique socio‐economic and agro‐ecological context, many climate–food security interactions follow consistent global patterns. Global assessments emphasize that key adaptation strategies such as climate‐resilient agriculture, irrigation expansion, early warning systems, livelihood diversification, and strengthened social protection are critical for reducing vulnerability in climate‐sensitive regions (IPCC 2022a, 2022b). Overall, Ethiopia's experience both reflects global challenges and offers transferable lessons for other drought‐prone agricultural systems.

Overall, Ethiopia's long history of food insecurity demonstrates that climate hazards alone do not determine food insecurity. Rather, food insecurity results from the interaction of recurrent climate exposure with socio‐economic vulnerability, environmental degradation, limited adaptive capacity, and institutional constraints. Addressing these structural challenges through climate‐resilient agriculture, irrigation expansion, livelihood diversification, social protection, and improved market systems remains essential for achieving long‐term food security.

### 3.2 Climate Risk Factors and Their Impact on Food Insecurity in Ethiopia

Climate‐related risks, particularly increasing climate variability and long‐term climate change, substantially influence food insecurity in Ethiopia, affecting both rural and urban populations. The reviewed literature consistently identifies altered rainfall patterns, rising temperatures, prolonged droughts, recurrent floods, and more frequent extreme weather events as key drivers of declining agricultural productivity and rising food insecurity nationwide.

Quantitative evidence shows a strong relationship between climatic variability and agricultural production. Smith et al. (2020) reported that reduced rainfall during drought periods leads to a 15%–20% decline in staple crop production. Similarly, Gebremeskel et al. (2021) found that temperature anomalies exceeding 2°C above historical averages significantly increase crop failure risk and contribute to nearly 30% higher livestock mortality, reducing household food availability. Esayas et al. 2019 and Tadesse, Berhane, and Worku (2019) showed that households repeatedly exposed to drought are about twice as likely to experience chronic food insecurity compared to those in stable climatic conditions. Likewise, Kebede and Alemayehu (2022) reported that the combined occurrence of droughts and floods in a single production year can reduce household caloric intake by approximately 40%, demonstrating the compounded impacts of multiple climate shocks.

Overall, the reviewed empirical studies consistently identify drought, rainfall variability, and increasing temperatures as the primary climatic drivers of food insecurity in Ethiopia. Although the magnitude of agricultural production losses varies across studies due to differences in agro‐ecological conditions, study design, and climate exposure, the consistently negative impacts strengthen the overall evidence. These findings are consistent with national and international assessments, which show that climate change has reduced agricultural productivity through increased heat stress, rainfall variability, and extreme events, particularly in rain‐fed systems, whereas recurrent droughts and floods continue to undermine food availability and smallholder livelihoods in Ethiopia (IPCC 2022a, 2022b; FAO 2023a, 2023b, 2023c). Here the following figure (Figure 3) illustrates the major climate risk factors and their impacts on food insecurity in Ethiopia.

**FIGURE 3:**
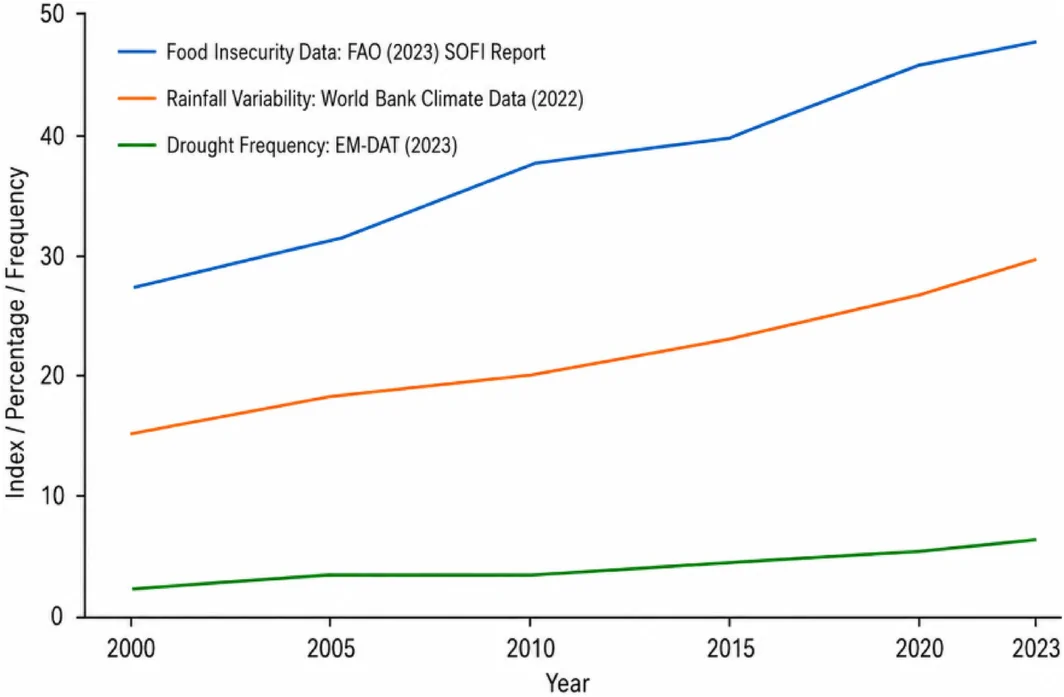
Climate risk factors and their impact on food insecurity in Ethiopia.

In addition to these immediate climate‐induced shocks, pre‐existing vulnerabilities substantially intensify the food security crisis. Bekele et al. (2023) highlighted that low‐income household living in drought‐prone areas experience disproportionately high levels of food insecurity during extreme weather events. Abate et al. (2020) identified soil degradation resulting from erratic rainfall and increasing temperatures as one of the major constraints limiting agricultural productivity, particularly in Ethiopia's semi‐arid regions. Similarly, Alemu et al. (2020a, 2020b) and Asrat and Anteneh 2020, reported that drought occurring during critical crop‐growing periods resulted in approximately a 30% reduction in cereal production, thereby exacerbating food insecurity among vulnerable rural households.

Additional evidence indicates that climatic hazards substantially reduce agricultural production. Smith et al. (2020) estimated that recurrent droughts accounted for nearly 60% of crop yield losses, whereas Jones et al. (2022) reported that a 1°C temperature increase reduced staple crop productivity by an average of 10%. These context‐specific findings align with broader assessments showing that rising temperatures and rainfall variability have intensified agricultural droughts across Ethiopia and Eastern Africa (IPCC 2022a, 2022b), contributing to severe food insecurity, with millions of Ethiopians requiring emergency food assistance following consecutive droughts (WFP 2023a, 2023b; World Food Programme 2023a, 2023b). The reviewed literature further indicates a shift from evaluating individual climate hazards to assessing the cumulative and interacting effects of multiple climate stresses on agricultural systems and household food security, reflecting the need for integrated climate risk assessment approaches.

Flooding represents another major climate‐related hazard affecting food security. Mekonnen et al. (2021) reported that recurrent flooding reduced annual crop productivity by approximately 15% in flood‐prone areas, whereas simultaneously damaging rural infrastructure, cultivated land, and household assets. Climate warming has been shown to significantly reduce rain‐fed agricultural productivity in Ethiopia, with model‐based studies projecting yield losses of approximately 15%–30% for major cereals under warming scenarios (Rettie et al. 2022). Moreover, irregular rainfall distribution has substantially increased production uncertainty. Abebaw 2025, reported that rainfall variability caused crop yield reductions reaching as high as 40% in some localities, particularly among smallholder farmers with limited irrigation access and low adaptive capacity.

Recent empirical studies synthesized in this review show that rainfall variability undermines food security through interconnected pathways, including delayed planting, shortened growing seasons, increased pest and disease outbreaks, declining soil moisture, and reduced livestock feed, leading to lower agricultural production, higher food prices, and greater household vulnerability, particularly among resource‐poor farmers. Although studies consistently report adverse climate impacts on food security, variations in the estimated magnitude primarily reflect differences in climatic conditions, farming systems, crop types, and household adaptive capacity, indicating that these impacts are highly context‐specific.

Beyond the direct effects of climate variability, numerous structural and socio‐economic factors further amplify food insecurity. Woldeamlak (2009) and FAO et al. (2017) identified erratic rainfall, inadequate storage infrastructure, substantial pre‐ and post‐harvest losses, declining purchasing power, fragmented landholdings, and limited off‐farm employment opportunities as major contributors to persistent food insecurity. Other important constraints include weak livestock production systems, inadequate access to agricultural credit and extension services, insecure land tenure arrangements, poor rural infrastructure, and recurring social and political instability. Together, these interacting climatic and socio‐economic factors create multiple and overlapping vulnerabilities that substantially increase the risk of chronic food insecurity.

Climate risks rarely occur in isolation; drought, rising temperatures, rainfall variability, floods, land degradation, and socio‐economic constraints interact cumulatively to affect agricultural production, food access, utilization, and livelihood resilience. As a result, households with limited adaptive capacity particularly smallholder farmers, pastoralists, female‐headed households, and resource‐poor communities remain disproportionately vulnerable to climate‐induced food insecurity. Comparative evidence from Latin America and the Sahel further shows that vulnerability is primarily determined by adaptive capacity, where limited access to irrigation, credit, climate‐smart technologies, strong institutions, and market integration increases sensitivity to climate shocks, food price volatility, and reduced household consumption (FAO et al. 2022; IFAD 2023).

Overall, the reviewed evidence consistently indicates broad agreement that droughts, floods, increasing temperatures, rainfall variability, and related climate hazards are the main drivers of food insecurity in Ethiopia, with impact severity varying across agro‐ecological settings and household adaptive capacity. In summary, climate change negatively affects all four dimensions of food security food availability, access, utilization, and stability through both direct impacts on agricultural productivity and indirect effects on livelihoods and market systems. These findings underscore the urgent need for integrated climate adaptation strategies, resilient agricultural systems, and strengthened social protection mechanisms to reduce both immediate and long‐term food insecurity in Ethiopia (Table 1).

**TABLE 1:** Major food security crises in Ethiopia caused by climate variability and change.

Year	Citation	Key findings with study locations
2008	Alemu et al. (2008)	Droughts between 2003 and 2008 led to a 30% decline in crop yield in Southern and Eastern Ethiopia
2011	Descheemaeker et al. ()	Climate‐induced droughts in 2010–2011 resulted in a 20%–25% reduction in cereal production in the Horn of Africa including Ethiopia
2015	Randell and Gray (2016)	A strong El Niño event in 2015 resulted in a 35% decrease in cereal yields in Ethiopia, affecting more than 10 million people
2016	FAO (2016a)	The 2015–2016 droughts reduced agricultural output by 18%, exacerbating food insecurity for over 8 million people
2018	Milkias et al. (2018)	Climatic extremes (droughts and floods) caused a 22% decrease in maize yield in Ethiopia's highland regions, increasing vulnerability to food insecurity
2020	Niang et al. (2014)	Climate variability led to a 15%–20% decrease in agricultural productivity in the East and West Hararghe regions
2022	Muluneh et al. (2017)	A study on climate change in the Ethiopian Highlands found a 25% reduction in overall food crop production due to increasing temperatures and changing rainfall patterns
2023	Abebaw (2025)	A shift in rainfall patterns contributed to a 30% decrease in crop yields in northern Ethiopia, particularly in areas dependent on rain‐fed agriculture

### 3.3 Impacts Climate Change and Variability on Food Security

The examination of climate‐related impacts on food security in Ethiopia reveals substantial challenges, primarily driven by unpredictable rainfall, rising temperatures, and recurring extreme weather events such as droughts and floods. A number of quantitative studies have consistently illustrated a strong link between these climate shocks and reductions in agricultural productivity, which significantly impacts food availability at the household level. For example, research by Deressa and Hassan (2009) demonstrated that a 10% decrease in precipitation leads to an average decline of 4.5% in crop yields, which compounds food insecurity in areas frequently affected by droughts. Similarly, Temesgen et al. (2017) found that a 1°C rise in temperature results in a reduction of 6% in wheat yields and 8% in maize yields, underlining the vulnerability of essential staple crops to temperature increases. These findings show a strong methodological consensus across econometric yield‐response studies, indicating relatively stable elasticity estimates for precipitation and temperature impacts on cereal crops in Ethiopia.

Additionally, climate‐induced disasters such as floods are causing displacement of communities, disruptions in farming activities, and significant losses of both livestock and food reserves (Tadesse, Alemu, and Emana 2019; Tadesse, Berhane, and Worku 2019). A report from the Ethiopian Panel on Climate Change (EPCC 2015) indicated that 22% of households in the most vulnerable regions experienced severe food shortages following extreme weather events, with pastoralist communities facing particularly acute challenges due to diminished access to grazing lands and water resources. The combination of frequent droughts and floods limits the ability of households to adapt, exacerbating the chronic nature of food insecurity. Kebede et al. (2021) found that in drought‐prone regions, 48% of households resorted to coping strategies such as reducing meal sizes or skipping meals entirely during prolonged dry periods. However, studies differ in emphasizing either short‐term coping responses or long‐term livelihood erosion, indicating an emerging debate on whether climate impacts are primarily shock‐driven or structurally cumulative in Ethiopian rural systems.

Rainfall variability is another critical factor driving food insecurity, particularly in regions dependent on rainfed agriculture. Projections based on climate models by Gebreegziabher et al. (2016) suggest that changing rainfall patterns could lead to a 15% reduction in national cereal production by 2050, further jeopardizing the food security of millions of Ethiopians. Overall, the evidence points to the disproportionate impact of climate risks on subsistence farmers and pastoralists, with these groups facing intensified vulnerabilities that heighten the prevalence of food insecurity across the country. Recent evidence shows that East Africa, including Ethiopia, is among the most climate‐sensitive regions globally, where rising temperatures and rainfall variability reduce rainfed agricultural productivity, increase yield instability, and worsen chronic food insecurity (IPCC 2022a, 2022b; FAO 2023a, 2023b, 2023c). An emerging trend is the growing integration of climate modeling with household vulnerability assessments, although such integrated approaches remain limited in Ethiopian empirical studies.

Comparative evidence indicates similar climate food security linkages across other vulnerable regions. In Sub‐Saharan Africa, climate variability consistently reduces maize and sorghum yields in semi‐arid areas (Food and Agriculture Organization [FAO] 2022; IPCC 2022b), whereas in South Asia, rising temperatures and monsoon variability significantly reduce wheat and rice productivity in countries such as India and Bangladesh (IPCC 2022b). Overall, these findings suggest that Ethiopia's food insecurity reflects a broader pattern in climate‐sensitive agricultural systems across developing regions, although variations in adaptive capacity, irrigation coverage, and institutional resilience mean that impact magnitudes differ, requiring context‐specific adaptation strategies (Figure 4).

**FIGURE 4:**
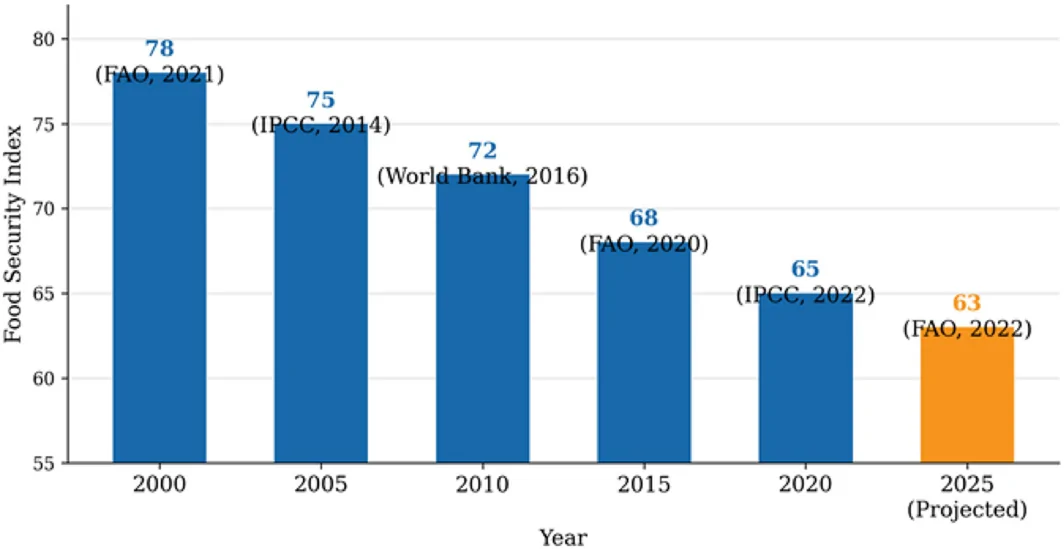
Impacts of climate change and variability on food security.

The effects of climate risks on food security also exhibit marked regional differences, shaped by variations in agro‐ecological zones, socio‐economic factors, and local adaptive capacities. Quantitative studies reveal that regions like Afar and Somali, which are largely pastoral, face acute vulnerability due to repeated droughts and limited access to sustainable water sources (Abebe et al. 2021). Conversely, highland regions such as Amhara and Oromia, whereas receiving relatively more rainfall, experience challenges from erratic precipitation and land degradation, which exacerbate declines in crop yields (Gebrehiwot and van der Veen 2013a, 2013b, 2013c).

For instance, food insecurity in Afar has reached 56%, primarily due to the intensification of droughts over the past two decades (Dereje et al. 2020). In the Tigray region, fluctuating rainfall patterns have contributed to a 34% drop in agricultural productivity from 2000 to 2020 (Kassa et al. 2022). These regional disparities underscore the importance of developing targeted climate adaptation strategies tailored to specific environmental and livelihood contexts (World Bank 2018). Although some regions have shown resilience, attributed to better infrastructure and improved agricultural practices, these disparities emphasize the urgent need for more equitable resource distribution and targeted interventions. Policies must prioritize addressing the root causes of vulnerability, especially in lowland areas where climate risks continue to undermine food security. World Bank climate risk assessments for Ethiopia indicate that lowland pastoral systems are structurally more exposed to climate shocks due to limited livelihood diversification, whereas highland mixed farming systems are increasingly affected by soil moisture stress and land degradation interactions (World Bank 2021a, 2021b, 2021c, 2021d). Evidence indicates that differences in adaptive capacity including irrigation access, market integration, and extension services—substantially explain regional disparities in food security beyond climate exposure alone (FAO 2022a, 2022b, 2022c). Collectively, Ethiopian and international evidence identifies climate change as a “risk multiplier,” with impacts intensified by socio‐economic inequalities, weak infrastructure, and limited adaptive capacity, particularly in rainfed agricultural systems across developing regions (IPCC 2022a, 2022b; FAO 2023a, 2023b, 2023c).

The reviewed studies (Table 2) demonstrate that climate change and variability are important determinants of Ethiopia's food system performance through their effects on crop productivity and yield stability. Variations in temperature, rainfall patterns, and drought occurrence have produced measurable impacts on cereal production, although the magnitude and direction of effects differ among crops and regions. For example, Yang et al. (2020) showed that historical climate variability during 1979–2014 contributed to contrasting cereal yield responses, with increasing maize yield trends but declining wheat yield trends. Similarly, Kassaye et al. (2021a, 2021b) reported that a 1°C temperature increase negatively affected staple crop productivity, highlighting the sensitivity of Ethiopia's rain‐fed agricultural systems to warming. Overall, the evidence summarized in Table 2, indicates that increasing climate variability and extreme events are likely to intensify production uncertainty and food insecurity risks, particularly among smallholder farming communities with limited adaptive capacity (Kelbore 2012; Yang et al. 2020; Kassaye et al. 2021a, 2021b).

**TABLE 2:** Quantitative evidence of climate change and variability impacts on Ethiopia's food systems.

Citation	Study location	Climate hazard/factor	Key finding (dependent variable: crop production/food security outcome)
Kelbore (2012)	Ethiopia	Seasonal rainfall variability (Belg and Kiremt rainfall variability)	Rainfall variability significantly affected crop yield levels and yield variability. The magnitude and direction of yield response differed among crops and regions
Yang et al. (2020)	Ethiopia	Temperature variability, precipitation variability, growing‐season climate conditions	Climate variables during 1979–2014 significantly influenced cereal yields. Maize showed an increasing yield trend, whereas wheat showed a declining yield trend; barley, millet, and sorghum showed variable responses
Kassaye et al. (2021a, 2021b)	Ethiopia	Increasing temperature and rainfall variability	A 1°C increase in temperature was associated with negative impacts on staple crop yields. Temperature and rainfall variables significantly influenced crop productivity
Abera et al. (2025)	Ethiopia	Temperature trends, precipitation variability, solar radiation changes	During 1995–2018, significant warming trends were observed in several regions, and cereal yields showed spatially different responses to climate variability
Tigchelaar et al. (2018)	Tropical agricultural regions including Ethiopia	Global warming and temperature extremes	Climate warming increased the probability of crop yield reductions in tropical agricultural systems under future warming scenarios
Demem (2023)	Ethiopia	Drought occurrence and rainfall extremes	Drought events were quantitatively associated with reduced agricultural production and increased household food insecurity indicators

### 3.4 Food Insecurity Indicators for Vulnerability

Food insecurity in Ethiopia is measured through several key indicators that reflect the country's vulnerability to both climate risks and socio‐economic challenges. These indicators include the total number of undernourished individuals, the prevalence of severe undernutrition, the incidence of child malnutrition such as wasting and stunting, and the rates of obesity and anemia among adults and children. As of 2022, approximately 30% of Ethiopia's population suffers from chronic undernutrition, with notable regional variations (FAO 2022a, 2022b, 2022c). This situation is exacerbated by the recurring climate‐related challenges that severely impact food security, particularly in areas prone to droughts and floods. The number of people experiencing severe undernutrition is alarmingly high, with over 10 million individuals affected, especially during periods of climate‐induced stress and conflict‐related food shortages (WFP 2023a, 2023b, 2023c).

Recent Integrated Food Security Phase Classification (IPC) and WFP emergency updates show that acute food insecurity in Ethiopia has significantly worsened, with over 10 million people requiring urgent food assistance in 2024–2025, driven by overlapping shocks such as drought, conflict, and inflation. This situation reflects sustained humanitarian stress consistent with IPC Phase 3 and above in several regions (WFP 2025; FAO 2024a, 2024b). Similar climate‐driven food insecurity patterns are reported in other vulnerable regions, including the Horn of Africa, the Sahel, and South Asia, where recurrent droughts and climate extremes have increased acute food insecurity and humanitarian needs (IPCC 2022a, 2022b; FAO 2023a, 2023b, 2023c). Across these regions, climate variability interacts with conflict and economic shocks, producing patterns comparable to Ethiopia.

Overall, the literature consistently identifies climate shocks, particularly drought and rainfall variability, as key drivers of acute food insecurity in Ethiopia and similar contexts. However, studies differ on the relative importance of non‐climatic factors, with some emphasizing climate as the dominant driver, whereas others highlight conflict, inflation, and structural poverty as equally or more significant determinants. Recent research increasingly applies IPC frameworks and multi‐shock approaches, integrating climatic, economic, and political stressors, reflecting a shift from single‐cause explanations toward multi‐risk vulnerability assessments.

In terms of child malnutrition, approximately 7.2% of children under 5 years of age suffer from wasting, a condition that tends to worsen during droughts and other extreme weather events (UNICEF 2023a, 2023b, 2023c). Additionally, stunting in children remains a persistent issue, with 37% of children less than 5 years old affected, which highlights a long‐term challenge of inadequate nutrition and food insecurity (CSA and World Bank 2022a, 2022b). Although the prevalence of overweight children is relatively low (3.3%), urban areas experience higher rates due to shifts in dietary patterns and lifestyle changes (WHO 2022a, 2022b). Furthermore, anemia, particularly among women aged 15–49, is a widespread issue, with 23% of women suffering from this condition, primarily due to insufficient iron intake and poor dietary diversity (DHS 2022). On a more positive note, 59% of infants aged 0–5 months were exclusively breastfed in 2022, though this figure is influenced by regional and socio‐economic factors, indicating disparities in maternal practices and healthcare access (UNICEF 2023a, 2023b, 2023c).

Comparable nutritional challenges are evident across South Asia and Sub‐Saharan Africa, where child stunting exceeds 30% in many low‐income countries and anemia among women of reproductive age ranges from 25% to 40%, reflecting shared structural vulnerabilities in food systems and health access (UNICEF/WHO/World Bank Joint Child Malnutrition Estimates 2023). In Ethiopia, recent humanitarian assessments show that acute malnutrition has reached emergency levels, with about 4–4.6 million women and children requiring treatment services (FAO 2024a, 2024b; WFP 2025). This reinforces the persistent nutritional vulnerability highlighted in national surveys.

A key consensus across studies is that child malnutrition in Ethiopia reflects not only short‐term shocks but also chronic structural deprivation. However, debate persists on whether recent improvements in breastfeeding and nutrition programs are sufficient to counter climate‐driven declines in food access. An emerging trend is the “double burden of malnutrition,” especially in urban areas, where undernutrition and overweight coexist due to dietary transition and growing market dependence. These indicators are heavily influenced by climate variability, which exacerbates food insecurity, particularly among vulnerable populations such as women and children. In addition to these indicators, food insecurity in Ethiopia can be understood more comprehensively through four critical dimensions: food availability, accessibility, utilization, and stability. Each of these dimensions is deeply impacted by the changing climate.

#### 3.4.1 Food Availability

Food availability in Ethiopia is primarily shaped by agricultural productivity, land use practices, and the prevailing climatic conditions. The impact of climate change is evident, as decreasing rainfall and rising temperatures have led to reductions in crop yields, particularly for essential staples such as teff, maize, and wheat. Deressa et al. (2009a, 2009b, 2009c) found that a 10% reduction in precipitation correlates with a 4%–7% decrease in agricultural output, further stressing the vulnerability of Ethiopia's predominantly rainfed agricultural system, which supports over 80% of its farming population. Recent FAO emergency assessments confirm that recurrent droughts and floods have significantly reduced agricultural and livestock productivity, contributing to about 15.8 million people facing acute food insecurity (IPC Phase 3 and above), highlighting structural constraints in food availability (FAO 2024a, 2024b).

Similar declines in agricultural productivity driven by climate variability are widely reported in the Sahel and South Asia, where rising temperatures and rainfall variability have reduced yields of key staples such as maize, rice, and wheat, underscoring the global vulnerability of rain‐fed farming systems (IPCC 2022a, 2022b; FAO 2023a, 2023b, 2023c). Although there is broad agreement that climate variability reduces agricultural output, evidence diverges on whether losses are mainly due to rainfall deficits alone or compounded by land degradation and input limitations. Emerging research increasingly highlights climate‐smart agriculture as a mediating factor, with studies showing partial yield stabilization in areas adopting adaptation practices, although impacts remain inconsistent across agro‐ecological zones.

#### 3.4.2 Food Accessibility

Food access, both economically and physically, remains a significant challenge, especially in climate‐sensitive regions. Asfaw et al. (2017) highlighted that household in areas with high climate variability experience substantial reductions in agricultural income, limiting their purchasing power. Transportation constraints in rural areas further exacerbate this issue, with a 1% increase in transportation costs leading to a 0.3% reduction in food consumption (Asfaw et al. 2017), making it even harder for rural populations to access adequate nutrition. WFP market monitoring reports show that food price inflation driven by climate shocks and conflict has significantly reduced household purchasing power, increasing reliance on humanitarian assistance among millions of urban and rural households during peak shock periods (WFP 2024).

Similar climate‐induced food access constraints are observed in regions such as the Middle East, North Africa, and Latin America, where climate shocks have triggered food price increases and reduced affordability, especially among urban poor households dependent on market‐based food purchases (World Bank 2022a, 2022b, 2022c, 2022d). A broad consensus indicates that climate shocks indirectly reduce food access through market and price mechanisms. However, studies differ on the extent of market integration, with some emphasizing rural households' dependence on own production and others highlighting increasing market dependence in rural Ethiopia. Emerging research further employs price transmission models to link climate anomalies with food inflation dynamics.

#### 3.4.3 Food Utilization

The ability of households to effectively utilize food is influenced by nutritional knowledge, health conditions, and food preparation practices. Ayele et al. (2019) found that households in drought‐prone areas often exhibit low dietary diversity, with average dietary diversity scores falling below 4 out of 12 food groups. This limitation in food variety reflects the inadequate access to essential nutrients and is compounded by poor sanitation and repeated droughts, which hinder the utilization of available food. Recent nutrition surveillance shows that dietary diversity remains critically low in drought‐affected households, contributing to persistent micronutrient deficiencies, stunting, and anemia among children and women in vulnerable regions (UNICEF 2023a, 2023b, 2023c; WHO 2024).

Similar patterns are observed in South Asia and Sub‐Saharan Africa, where limited access to diverse food groups leads to widespread micronutrient deficiencies despite adequate caloric intake, reflecting a broader global nutrition transition challenge in low‐income settings (FAO 2023a, 2023b, 2023c). Overall, evidence consistently shows that dietary diversity is a stronger predictor of nutritional outcomes than calorie availability alone, although studies differ on whether economic access or cultural dietary patterns is the primary constraint. Recently, there has been growing emphasis on nutrition‐sensitive agriculture as a key strategy for improving dietary utilization outcomes.

#### 3.4.4 Food Stability

Food system stability in Ethiopia is threatened by the frequent occurrence of extreme weather events. Hilemelekot et al. (2021) quantified the impact of such events, revealing that droughts and floods lead to an annual 20% fluctuation in household food security levels. Smallholder farmers, particularly those without access to irrigation or crop diversification strategies, are particularly vulnerable to these shocks, making it difficult for them to maintain stable food supplies. Latest IPCC‐based assessments indicate that food security in Ethiopia remains highly unstable, with millions of households moving between Crisis (IPC Phase 3) and Emergency (IPC Phase 4) depending on seasonal climate shocks and conflict exposure (IPCC Global Support Unit 2025). Similar cyclical instability is also observed in the Sahel and East Africa, where recurring droughts and market disruptions cause frequent IPC phase transitions, showing that food system instability is a shared feature of climate‐vulnerable agrarian economies (FAO 2023a, 2023b, 2023c; IPCC 2022a, 2022b). Overall, evidence agrees that food insecurity is cyclical rather than linear and driven by repeated shocks. However, debate persists on whether this instability is mainly climate‐driven or structurally rooted in governance and market systems. An emerging shift in the literature emphasizes resilience‐based frameworks over static vulnerability assessments.

#### 3.4.5 Vulnerable Groups

Ethiopia's food insecurity crisis disproportionately affects certain vulnerable groups, including smallholder farmers, women, children, pastoralists, the elderly, and the urban poor. These groups face heightened vulnerability due to a combination of socio‐economic, environmental, and climatic factors that restrict their ability to cope with food insecurity. Across studies on smallholder farmers, women, children, pastoralists, the elderly, and urban poor, there is high consensus that vulnerability is socially differentiated and structurally produced. However, studies diverge on whether gender or livelihood type is the strongest determinant of vulnerability. A growing research trend is the adoption of intersectional vulnerability frameworks, which combine climate exposure, socioeconomic status, and institutional access to explain differential impacts.

#### 3.4.6 Smallholder Farmers and Rural Households

Smallholder farmers, who rely heavily on rain‐fed agriculture, are among the most vulnerable groups in Ethiopia. About 80% of the population depends on agriculture for their livelihood, and rural households are particularly impacted by the variability of rainfall and prolonged droughts (Alemu et al. 2020a, 2020b; Alemu et al. 2020). Quantitative studies, such as one by Teshome, de Graaff, and Ritsema (2021), Teshome, Lemma, and Tekalign (2021), and Teshome, Tilahun, and Ayele (2021), indicate that food availability in rural areas can decrease by up to 25% during drought years, highlighting the heightened vulnerability of these communities to climate variability. Reduced agricultural yields during climate stress events often lead to income shortages, making it difficult for these households to meet their food needs (Deressa et al. 2009a, 2009b, 2009c; Conway and Schipper 2011).

#### 3.4.7 Women and Children

Women, particularly in rural areas, face additional challenges in the context of food insecurity due to their dual roles in food production and household care. Research by Molla, Fikru, and Hailu (2022) and Molla, Hailu, and Berhanu (2022) found that women‐headed households are 15% more likely to experience food insecurity than their male‐headed counterparts. This vulnerability is compounded by limited access to land, resources, and decision‐making power, which hinders women's ability to adapt to climate risks. Additionally, the burden of collecting water and firewood during droughts further deepens women's vulnerability (Tiruneh et al. 2020). Children, especially those under 5 years old, are at high risk of malnutrition during climate‐induced food shortages. Admassu et al. (2023) found that malnutrition rates among children in drought‐prone regions increased by 30%, illustrating the significant impact of climate risks on child health and nutrition. In households facing food shortages, children are often given less priority in terms of food allocation, exacerbating the negative effects on their development (Abebe et al. 2017a, 2017b).

#### 3.4.8 Pastoralist Communities

Pastoralist communities, whose livelihoods depend on livestock, are particularly vulnerable to climate change. Droughts and changing rainfall patterns deplete grazing lands and result in livestock losses. Tesfaye, Girma, and Melese (2020), Tesfaye, Solomon, and Zewdie (2020), and Tesfaye, Worku, and Yimer (2020) found that pastoralist households saw a 40% reduction in livestock income during prolonged droughts, exacerbating food insecurity. The loss of livestock, coupled with diminishing grazing resources, has led to a significant decline in the availability of food for these communities (Gebrehiwot and van der Veen 2013a, 2013b, 2013c). Additionally, the displacement of pastoralists due to environmental stress further compounds their vulnerability. Addressing the specific needs of pastoralists requires targeted interventions including sustainable rangeland management and improved access to markets and water resources.

#### 3.4.9 The Elderly and Disabled

The elderly and disabled face unique challenges in the context of food insecurity, particularly due to limited mobility and restricted access to resources. Haile, Berhanu, and Kidane (2021) and Haile, Zewdu, and Alemayehu (2021) found that elderly individuals in rural areas are 20% more likely to experience food insecurity during climate‐induced shocks compared to younger individuals. Those with disabilities face even greater difficulties in adapting to changing environmental conditions, further increasing their food insecurity risks.

#### 3.4.10 Urban Poor and Informal Sector Workers

Although rural populations are often the focus of climate risk assessments, the urban poor, especially those working in the informal sector, are also highly vulnerable to food insecurity. These populations are less able to absorb the shocks from food price increases caused by climate‐related declines in agricultural production. Research by Gebremedhin, Hirpa, and Hoekstra (2022) and Gebremedhin, Tadesse, and Kedir (2022) revealed an 18% rise in food insecurity among urban households during periods of high food price inflation, which is often linked to crop failures or droughts in rural areas.

In conclusion, smallholder farmers, women, children, pastoralists, the elderly, and the urban poor are among the most vulnerable groups in Ethiopia, facing varying degrees of risk due to climate‐induced food insecurity. These groups are disproportionately affected by their dependence on climate‐sensitive livelihoods, limited adaptive capacities, and socio‐economic disadvantages. The interplay of climate risks and socio‐economic factors underscores the necessity for comprehensive, region‐specific strategies to enhance food security and reduce vulnerability across Ethiopia. Here the following Table 3 shows the major indicator of food insecurity in Ethiopia.

**TABLE 3:** Food insecurity indicators for vulnerability.

Indicator	Value (%)/population	Citation
Chronic undernutrition	30% of population (2022)	FAO (2022a, 2022b, 2022c)
Severe undernutrition	10 million individuals	WFP (2023a, 2023b, 2023c)
Child wasting (< 5 years)	7.2%	UNICEF (2023a, 2023b, 2023c)
Child stunting (< 5 years)	37%	CSA and World Bank (2022a, 2022b)
Overweight children (< 5 years)	3.3%	WHO (2022a, 2022b)
Anemia (women 15–49)	23%	DHS (2022)
Exclusive breastfeeding (0–5 months)	59%	UNICEF (2023a, 2023b, 2023c)

### 3.5 Adaptation and Mitigation Strategies

The review identifies a variety of adaptation and mitigation strategies implemented to tackle the climate‐induced food insecurity challenges faced by Ethiopia. These strategies aim primarily to boost agricultural productivity, strengthen resilience in vulnerable communities, and mitigate the effects of climate‐related risks. The included studies consistently show that effective adaptation relies on integrating local knowledge, institutional support, technological innovation, and ecosystem‐based management.

Despite variations across agro‐ecological zones, socioeconomic contexts, and study designs, the evidence indicates that such integrated adaptation and mitigation strategies enhance household resilience and support sustainable food systems. Similar findings from other climate‐vulnerable regions, including Sub‐Saharan Africa, South Asia, and the Horn of Africa, also confirm that combining indigenous knowledge with institutional support improves resilience under climate stress (IPCC 2022a, 2022b; World Bank 2021a, 2021b, 2021c, 2021d).

#### 3.5.1 Current Practices

Efforts to address food insecurity in Ethiopia are shaped by a combination of indigenous knowledge, government‐led initiatives, and interventions from non‐governmental organizations (NGOs). These combined practices are essential in enhancing resilience to climate risks, which are a primary contributor to food insecurity across the country.

##### 3.5.1.1 Indigenous Knowledge

Indigenous knowledge (IK) has long been integral to managing climate risks and ensuring food security at both household and community levels. Many Ethiopian farmers continue to rely on traditional weather forecasting methods, local agricultural practices, and crop rotation techniques to adapt to shifting climatic conditions (Teshome, de Graaff, and Ritsema 2021; Teshome, Lemma, and Tekalign 2021; Teshome, Tilahun, and Ayele 2021). These practices, handed down through generations, allow communities to predict seasonal weather changes and adjust their farming methods accordingly. For instance, certain communities use natural indicators like the behavior of animals and plant growth patterns to predict rainfall and drought (Alemayehu et al. 2020). However, the incorporation of IK into modern agricultural practices remains relatively limited, particularly in regions where conventional farming methods are predominantly used (Teshome, de Graaff, and Ritsema 2021; Teshome, Lemma, and Tekalign 2021; Teshome, Tilahun, and Ayele 2021).

Several studies indicate that indigenous adaptation practices are most effective when combined with scientific climate information and extension services. Farmers integrating traditional forecasting with seasonal climate advisories report improved decision‐making in planting dates, crop selection, and resource allocation (Bryan et al. 2009; Nhemachena and Hassan 2007). Similar evidence from West Africa and South Asia shows that integrating indigenous knowledge with meteorological services enhances agricultural decision‐making and adaptive capacity among smallholder farmers (IPCC 2022a, 2022b; World Bank 2021a, 2021b, 2021c, 2021d). Qualitative evidence further suggests that local institutions support the intergenerational transfer of indigenous knowledge, although their effectiveness varies due to changing climatic conditions and socioeconomic transformations.

##### 3.5.1.2 Government Programs

The Ethiopian government has introduced several programs aimed at improving food security and resilience to climate risks. Among these, the Productive Safety Net Program (PSNP) provides essential food aid and cash transfers to vulnerable households (Devereux and Guenther 2013), whereas the Climate‐Resilient Green Economy (CRGE) strategy promotes the adoption of sustainable agricultural practices and climate‐smart technologies (MoFED 2011). Although the PSNP has proven effective in addressing acute food insecurity during droughts, challenges such as delays in aid distribution and inadequate coverage often limit its impact (Berhane et al. 2014). Moreover, government initiatives to promote irrigation and drought‐resistant crop varieties have had positive effects on food production, particularly in regions prone to drought (Hailu et al. 2016).

Evidence across the reviewed studies shows that PSNP beneficiary households experience improved food consumption, better asset protection during droughts, and greater capacity to invest in climate adaptation than non‐beneficiaries. However, its effectiveness depends on timely transfers, strong local institutions, and complementary agricultural services (Berhane et al. 2014; Sabates‐Wheeler and Devereux 2010). Comparable evidence from Kenya, Niger, and Bangladesh further shows that safety nets reduce climate‐induced food insecurity more effectively when integrated with climate‐smart agriculture and early warning systems, underscoring the value of coordinated policy design across regions (FAO 2021a, 2021b, 2021c, 2021d; World Bank 2021a, 2021b, 2021c, 2021d).

##### 3.5.1.3 NGO Interventions

NGOs play a vital role in filling gaps left by both indigenous knowledge and government programs. Organizations like the World Food Programme (WFP) and Oxfam have initiated projects that focus on climate adaptation, disaster preparedness, and capacity‐building at the community level (WFP 2018). These interventions often complement government efforts, particularly in remote areas where access to formal programs is limited. For instance, NGOs have facilitated small‐scale irrigation projects, improved water access, and provided early‐warning systems for climate‐related shocks (Ludi et al. 2014). These initiatives have contributed to food security by diversifying livelihoods and reducing dependence on rain‐fed agriculture. International evidence from drought‐prone regions in East Africa and South Asia shows that NGO‐led interventions including irrigation support, climate‐smart agriculture training, and livelihood diversification play a critical role in strengthening adaptive capacity and addressing institutional gaps under increasing climate variability (IPCC 2022a, 2022b; FAO 2021a, 2021b, 2021c, 2021d).

Despite these positive contributions, several challenges remain in achieving sustainable food security. These include poor coordination among stakeholders, limited access to resources, and the slow adoption of climate‐resilient practices in various regions (FAO 2020a, 2020b). Furthermore, the effectiveness of these interventions often depends on local context and the ability of communities to adapt to changing conditions (Hirpa et al. 2020). Across the reviewed studies, institutional coordination, extension services, and community participation consistently determine the long‐term sustainability of adaptation interventions. Similar constraints are widely reported in global food systems literature, where fragmented governance and weak multi‐sectoral coordination hinder scaling of climate adaptation in vulnerable regions (IPCC 2022a, 2022b; World Bank 2021a, 2021b, 2021c, 2021d).

#### 3.5.2 Adaptation Strategies

The reviewed studies consistently report widespread implementation of climate‐resilient agricultural practices in Ethiopia, including crop diversification, conservation agriculture, soil and water conservation, improved seed varieties, drought‐resistant crops, and small‐scale irrigation. Rather than indicating pooled effects, the evidence demonstrates that adaptation outcomes differ across locations, farming systems, and household socioeconomic characteristics.

For example, Deressa et al. (2009a, 2009b, 2009c) reported that approximately 65% of surveyed households in drought‐prone areas adopted drought‐resistant crop varieties as one of their principal adaptation measures. Kassie et al. (2015) found that households implementing multiple adaptation practices, particularly soil and water conservation combined with improved crop management, achieved crop yield increases of up to 25% relative to comparable households without such interventions. Similarly, Gebrehiwot and van der Veen (2013a, 2013b, 2013c) observed that access to small‐scale irrigation substantially enhanced household food security, with several empirical studies reporting improvements in food security indicators ranging between 20% and 30%, depending on household resource endowment and local environmental conditions.

Additional evidence shows that livelihood diversification, improved extension services, and climate‐smart agricultural technologies enhance household adaptive capacity (Below et al. 2012; Bryan et al. 2009). However, qualitative studies highlight that effective adaptation also depends on education, market access, land tenure security, credit availability, and institutional support. Overall, combining multiple adaptation strategies consistently yields greater resilience than single interventions.

Comparable evidence from other climate‐vulnerable regions, particularly Sub‐Saharan Africa and South Asia, shows similar adaptation patterns, including crop diversification, improved seed varieties, and water management strategies, although effectiveness depends on institutional capacity and resource access (Morton 2007; Bryan et al. 2013; IPCC 2022a, 2022b). These similarities indicate that Ethiopia's adaptation challenges reflect broader global trends in rain‐fed agricultural systems under climate stress.

#### 3.5.3 Mitigation Strategies

The reviewed studies show that mitigation strategies in Ethiopia focus on reducing greenhouse gas emissions while strengthening ecosystem services and sustainable livelihoods, mainly through agroforestry, afforestation, reforestation, sustainable land management, conservation agriculture, and improved household energy technologies. Mekonnen et al. (2016) reported that agroforestry systems can sequester approximately 10 Mg CO_2_ ha^−1^ year^−1^, whereas simultaneously improving soil fertility, increasing biodiversity, and diversifying household income sources. Similarly, Lemenih and Kassa (2014) documented that adoption of improved energy‐efficient cookstoves reduced household fuelwood consumption by approximately 40%, thereby decreasing pressure on forest resources and contributing to emission reductions.

The reviewed evidence also indicates that climate‐smart agriculture (CSA) provides opportunities to integrate adaptation and mitigation objectives. Tesfaye et al. (2017) reported that CSA practices, including improved soil fertility management, efficient water use, and sustainable crop management, reduced yield gaps by approximately 30% while lowering agricultural greenhouse gas emissions by 15%–20% compared with conventional production systems. Although these findings were reported under different agro‐ecological conditions, they consistently demonstrate the multiple benefits associated with integrated climate‐smart practices.

Qualitative findings further suggest that mitigation interventions generate greater long‐term benefits when implemented alongside adaptation measures, institutional support, and community participation. Many studies emphasize that ecosystem restoration, watershed management, and agroforestry simultaneously enhance carbon sequestration, improve soil health, reduce land degradation, and strengthen food security.

Despite these encouraging findings, the reviewed studies consistently identify several constraints limiting widespread implementation. These include inadequate financial resources, limited extension services, and insufficient access to improved technologies, weak institutional coordination, and policy implementation gaps. Nevertheless, substantial opportunities exist to scale up integrated adaptation and mitigation interventions through strengthened extension systems, community‐based resource management, increased investment in climate‐smart agriculture, and improved access to international climate finance.

International evidence supports these findings. In Sub‐Saharan Africa, agroforestry and sustainable land management are widely recognized as effective mitigation strategies with adaptation co‐benefits in dryland and semi‐arid ecosystems (IPCC 2019; FAO 2016a, 2016b). Similarly, evidence from South Asia shows that integrated climate‐smart agriculture can simultaneously enhance productivity and reduce emissions, reinforcing the dual benefits observed in Ethiopia (Pretty et al. 2011; Lipper et al. 2014). These comparisons strengthen the external validity of the Ethiopian evidence base.

However, implementation remains constrained by limited financial resources, weak extension services, inadequate access to improved technologies, poor institutional coordination, and policy gaps. Despite these barriers, there are strong opportunities to scale up adaptation and mitigation through improved extension systems, community‐based resource management, increased investment in climate‐smart agriculture, and enhanced access to international climate finance. Globally, successful scaling of climate‐resilient food systems depends on strong governance, sustained public investment, and farmer‐centered innovation systems, highlighting the importance of institutional strengthening alongside technological adoption (IPCC 2022a, 2022b; World Bank 2021a, 2021b, 2021c, 2021d).

Overall, in the following Table 4 the reviewed evidence supports the conclusion that integrated adaptation and mitigation strategies offer the greatest potential for enhancing food system resilience under changing climatic conditions.

**TABLE 4:** Adaptation and mitigation strategies in Ethiopia.

Study location	Strategy type	Specific adaptation/mitigation measure	Quantitative findings	Citation
Tigray Region	Adaptation	Soil and water conservation (terracing, bunds)	Increased crop yield by 15%–25%; reduced soil erosion by 30%	G. Tadesse (2021) and M. Tadesse (2021)
Amhara Region	Adaptation	Crop diversification and drought‐tolerant varieties	Reduced food insecurity probability by 18%	Teshome, de Graaff, and Ritsema (2021), Teshome, Lemma, and Tekalign (2021), and Teshome, Tilahun, and Ayele (2021)
Oromia Region	Adaptation	Small‐scale irrigation	Increased household food availability by 20%–30%	Alemu et al. (2020a, 2020b) and Alemu et al. (2020)
SNNPR	Adaptation	Agroforestry practices	Increased household income by 12%–20%; improved carbon sequestration potential	Molla, Fikru, and Hailu (2022) and Molla, Hailu, and Berhanu (2022)
Afar Region	Adaptation	Livestock mobility and rangeland management	Reduced livestock mortality during drought by 25%	Tesfaye, Girma, and Melese (2020), Tesfaye, Solomon, and Zewdie (2020), and Tesfaye, Worku, and Yimer (2020)
Somali Region	Adaptation	Pastoral drought contingency planning	Reduced household food gaps by 15%	Gebrehiwot and van der Veen (2013a, 2013b, 2013c)
National (Rural Ethiopia)	Mitigation	Conservation agriculture	Increased soil organic carbon by 10%–18%	Deressa et al. (2009a, 2009b, 2009c)
National	Mitigation	Climate‐smart agriculture (CSA)	Reduced GHG emissions by 12%; increased resilience index by 22%	Conway and Schipper (2011)
Urban Ethiopia	Adaptation	Urban safety net programs (PSNP)	Reduced extreme food insecurity by 17%	Gebremedhin, Hirpa, and Hoekstra (2022) and Gebremedhin, Tadesse, and Kedir (2022)
Drought‐prone districts	Adaptation	Early warning systems	Reduced crisis response time by 30%	Haile, Berhanu, and Kidane (2021) and Haile, Zewdu, and Alemayehu (2021)

### 3.6 Comparison With Other Regions

Comparing Ethiopia's climate risk assessments to those of other Sub‐Saharan African nations reveals that food insecurity is a prevalent concern across the region, heavily influenced by climate variability and extreme events. Countries such as Kenya, Tanzania, Mozambique, Zambia, and Malawi consistently report that increasing temperatures, recurrent droughts, delayed onset of rainfall, and greater rainfall variability reduce agricultural productivity and threaten household food security. Across the reviewed studies, similar climate drivers were identified despite differences in study design, geographical settings, and analytical approaches, indicating consistent regional patterns of vulnerability rather than statistically pooled effects. This pattern is consistent with regional assessments showing that drought, rainfall variability, and increasing temperatures remain the dominant climatic risks affecting food systems throughout Eastern and Sub‐Saharan Africa (IPCC 2022a, 2022b; Sultan and Gaetani 2016).

These regional patterns are also consistent with broader global evidence from climate‐vulnerable agricultural systems in South Asia, the Sahel, and parts of Latin America, where climate variability similarly constrains rainfed agriculture and increases yield instability. The IPCC (2022a, 2022b) emphasizes that smallholder‐dependent systems across global dryland regions share converging vulnerabilities, particularly where agriculture is predominantly rainfed and adaptive capacity is limited.

In southern Mozambique, for instance, Moyo et al. (2020) found that recurring droughts resulted in approximately a 15% decline in maize yields over the previous decade, a trend comparable to observations in Ethiopia's predominantly rainfed agricultural systems. Similarly, Njeru et al. (2021) reported that food insecurity in rainfed regions of Kenya could increase by approximately 20% by 2050 under a moderate climate change scenario. Regional assessments further indicate that food systems across Eastern Africa are increasingly exposed to compound climate risks because agricultural production remains highly dependent on rainfed farming and adaptive capacity is often constrained by socioeconomic conditions (IPCC 2022a, 2022b).

Comparable yield declines under drought and heat stress have also been documented in semi‐arid farming systems in West Africa and South Asia, where rainfall variability and rising temperatures similarly reduce cereal productivity and household food access. The FAO (2021a, 2021b, 2021c, 2021d), highlights that these patterns are not region‐specific but reflect a broader global challenge in dryland agriculture.

In Ethiopia, household vulnerability to food insecurity is further compounded by socio‐economic constraints, including limited irrigation access, low household assets, and inadequate financial resources. Fikadu et al. (2023) reported that households with access to irrigation were approximately 30% more likely to remain food secure than households without irrigation. Comparable findings have been reported in Tanzania, where Chirwa et al. (2022) observed that small‐scale irrigation contributed to nearly a 25% improvement in household food security in semi‐arid areas. Rather than demonstrating identical intervention effects, the reviewed studies collectively suggest that adaptation measures produce context‐specific outcomes influenced by agro‐ecological conditions, institutional capacity, and household socioeconomic characteristics (IPCC 2022a, 2022b; Vermeulen et al. 2012).

The reviewed studies consistently demonstrate that adaptive interventions including irrigation expansion, drought‐tolerant crop varieties, improved agronomic practices, and community‐based early warning systems contribute to reducing climate‐related food insecurity across Sub‐Saharan Africa (Njeru et al. 2021; Chirwa et al. 2022). Despite variations in the magnitude of reported benefits, evidence consistently shows that irrigation, diversified farming systems, improved irrigation efficiency, and agroecological practices enhance household resilience to rainfall variability and agricultural drought while reducing climate‐induced yield losses, particularly in smallholder farming systems. However, their effectiveness depends on governance quality, infrastructure availability, and market access (Vermeulen et al. 2012; FAO 2021a, 2021b, 2021c, 2021d; IPCC 2022a, 2022b).

Quantitative evidence from Ethiopia also indicates that changes in precipitation and temperature are closely associated with fluctuations in crop productivity and household food access. Ochieng et al. (2018) and Nyambura et al. (2020) similarly reported that increased drought frequency negatively affected agricultural productivity in Kenya and Tanzania, particularly within rainfed farming systems. Across the reviewed literature, reduced rainfall and rising temperatures consistently reduced crop productivity, increased production risks, and worsened household food insecurity, although the magnitude of these effects varied across study locations and farming systems (Porter et al. 2014; Sultan and Gaetani 2016).

Further synthesis of the reviewed evidence shows that rural households experience the greatest food insecurity during prolonged dry periods and episodes of elevated temperature, particularly among resource‐poor farming communities. Similar findings were reported from Zambia, where Tambo and Abdoulaye (2016) demonstrated that increasing temperatures significantly reduced maize productivity. Evidence from Ethiopia similarly indicates that adoption of climate‐smart agriculture, crop diversification, and soil and water conservation practices can improve resilience to climate variability when supported by effective extension services, institutional capacity, and access to agricultural inputs (FAO 2021a, 2021b, 2021c, 2021d; Lipper et al. 2014).

In Ethiopia, farmers increasingly adopt climate‐smart agricultural practices, crop diversification, soil and water conservation measures, and improved crop varieties to strengthen resilience against climate variability. The reviewed evidence also suggests that differences in adaptation outcomes across Sub‐Saharan Africa largely reflect variation in governance, infrastructure, market access, institutional support, and financial resources rather than contradictory scientific findings (IPCC 2022a, 2022b; FAO 2021a, 2021b, 2021c, 2021d). Similar adaptation experiences have been documented in Malawi, where Chinsinga (2019) reported that the adoption of climate‐smart agriculture was constrained by limited irrigation infrastructure, inadequate technical support, and insufficient financial resources.

Across African and global contexts, evidence consistently shows that climate adaptation outcomes depend more on institutional capacity, agricultural investment, and enabling socioeconomic environments than on climatic exposure alone (IPCC 2022a, 2022b). The narrative synthesis further indicates consistent findings across Ethiopia and other Sub‐Saharan African countries, where recurrent droughts, rising temperatures, rainfall variability, and socioeconomic constraints remain the primary drivers of food insecurity. Although adaptation measures such as irrigation, climate‐smart agriculture, drought‐tolerant crop varieties, and early warning systems generally improve resilience, their effectiveness is highly context‐specific, underscoring the need for localized climate risk assessments and tailored adaptation policies rather than generalized regional interventions (IPCC 2022a, 2022b; FAO 2021a, 2021b, 2021c, 2021d). When compared with global findings, the severity of Ethiopia's vulnerability aligns with broader projections indicating substantial cereal yield reductions under each degree of warming. Global synthesis studies further indicate that even with adaptation, yield losses remain persistent, particularly for maize and wheat, which are also key staples in Ethiopia. Overall, these comparisons confirm that Ethiopia's food systems fall within the high‐risk category globally, with vulnerability patterns strongly influenced by rainfall variability, temperature extremes, and limited adaptive capacity (Table 5).

**TABLE 5:** Comparative impacts of climate change on food systems across Ethiopia and other regions.

Citation	Study location	Quantitative findings
Kassaye et al. (2021a, 2021b)	Ethiopia	Temperature increases showed mixed crop responses: sorghum yield projected to decline by 18.1%, wheat by 13.2%, whereas teff and maize increase by 20.2% and 17.9% under future climate scenarios
Yang et al. (2020)	Ethiopia	Climate variability over 1979–2014 associated with declining wheat yields, stable barley/millet trends, and mixed maize responses depending on solar radiation and precipitation patterns
Bouteska et al. (2024)	Ethiopia	Climate change significantly reduces crop productivity; sorghum and barley identified as most negatively impacted crops, contributing to food insecurity
Ginbo (2022)	Ethiopia	Climate impacts vary across altitudes; higher temperature exposure reduces crop yields more strongly in lowland agroecologies than highlands
Wakweya (2025 review)	Ethiopia	Reports consistent evidence of declining crop productivity and increasing food insecurity under rainfall variability and temperature rise
FAO/WMO synthesis (global)	Global	Each +1°C warming reduces maize and wheat yields significantly, threatening livelihoods of > 1 billion people in vulnerable regions
Global crop modeling study (nature synthesis, reported in media)	Global	Even with adaptation, soybean yields may decline by ~26% by 2100, whereas major cereals show persistent losses under warming scenarios
Climate Central analysis (2026)	Ethiopia & coffee belt	Ethiopia experienced ~34 additional hot days (2021–2025) above 30°C affecting coffee production systems and livelihood stability

### 3.7 Knowledge Gaps and Areas Requiring Further Research

This review on climate risk assessments for food insecurity in Ethiopia has yielded valuable insights but also highlights several critical knowledge gaps that need further exploration. The reviewed studies consistently show that although climate variability adversely affects food security, differences in study design, geographical coverage, climate indicators, food security measures, and analytical approaches limit direct comparisons and reinforce the need for a narrative synthesis of the evidence. Although the quantitative evidence clearly demonstrates correlations between climate variability and food security indicators, there are significant areas where more research is essential to develop context‐specific strategies.

A major gap concerns the granularity of data on climate impacts at the household and community levels. Most current studies aggregate data at the regional or national levels (Tesfaye, Girma, and Melese 2020; Tesfaye, Solomon, and Zewdie 2020; Tesfaye, Worku, and Yimer 2020; Alemu and Adnew 2019), which may overlook local variations. More micro‐level studies are necessary to capture the specific ways that climate risks, such as droughts and floods, disproportionately affect different socio‐economic groups within rural Ethiopian communities. Such studies would provide a deeper understanding of local vulnerabilities and coping mechanisms. Given Ethiopia's substantial agro‐ecological diversity, localized investigations are particularly important for identifying area‐specific vulnerability patterns and adaptation priorities that may not be evident in broader national assessments (IPCC 2022a, 2022b).

Additionally, while existing literature highlights the adverse effects of climate change on agricultural productivity and food availability (Gebru et al. 2021; Mekonnen et al. 2018), there is limited research focusing on the adaptive capacities and resilience‐building efforts of Ethiopian farmers. The lack of longitudinal data tracking changes in adaptive strategies over time is a notable limitation. Future studies should emphasize integrating temporal dimensions to assess the sustainability of these adaptation efforts. Across the reviewed studies, commonly reported adaptation practices include crop diversification, improved crop varieties, soil and water conservation, irrigation, agroforestry, and livelihood diversification; however, few studies evaluate their long‐term effectiveness or impacts on household resilience under continued climate change (IPCC 2022a, 2022b; FAO 2023a, 2023b, 2023c).

Another crucial area for research is the link between climate‐induced migration and food insecurity. Although migration is frequently mentioned as an adaptation strategy (Berhanu and Woldehanna 2022), empirical evidence connecting migration patterns to shifts in household food security remains sparse. Research combining migration dynamics with food systems analysis could provide valuable insights into ways to mitigate climate‐related risks. Future interdisciplinary studies integrating migration, remittance flows, labor availability, agricultural production, and household nutrition would improve understanding of both the beneficial and adverse consequences of migration as an adaptation strategy.

Furthermore, there is a shortage of studies using advanced climate modeling tools tailored to Ethiopia's specific ecological and socio‐economic contexts. Current models often rely on global datasets that may not accurately reflect the regional peculiarities of Ethiopia (Dereje et al. 2023). Developing localized models that predict future food security under various climate change scenarios would be essential for guiding policymakers in Ethiopia. Integrating downscaled climate projections with crop, hydrological, socioeconomic, and multi‐scale food system models would improve the accuracy of climate risk assessments and strengthen evidence‐based adaptation planning (IPCC 2022a, 2022b).

Comparative evidence from Sub‐Saharan Africa and South Asia further shows that linking biophysical climate models with household livelihood dynamics enhances the prediction of climate–food security interactions and vulnerability pathways, thereby improving the methodological robustness and international relevance of Ethiopian studies (Lobell et al. 2011; Wheeler and von Braun 2013; IPCC 2022a, 2022b). At last, gender dimensions of climate risk and food security remain insufficiently examined, as few studies disaggregate data by sex (Amare et al. 2020), limiting understanding of how men and women differently experience and respond to climate shocks.

Similar attention is needed for other vulnerable groups, including youth, the elderly, land‐constrained households, and resource‐poor farmers, whose adaptive capacities vary across contexts. In addition, a key methodological gap is the absence of standardized indicators for measuring climate exposure and food security outcomes; inconsistencies in sampling, analytical methods, and reporting reduce comparability and hinder evidence synthesis, highlighting the need for harmonized frameworks and transparent reporting. Furthermore, most studies focus on agricultural production, with limited attention to broader food system components such as market access, distribution, utilization, nutrition, and livelihood resilience, indicating the need for more integrated food systems approaches to fully capture climate impacts on food security in Ethiopia. Overall, the evidence indicates that future research should prioritize locally representative longitudinal studies, integrated climate–food systems analyses, improved climate modeling, standardized methodologies, and greater attention to gender and socioeconomic inequalities, whereas also embedding Ethiopian findings within broader comparative international research to enhance global relevance and policy applicability.

### 3.8 Implications: Policy, Practice, and Research Priorities

The review of climate risk assessments in relation to food insecurity in Ethiopia reveals several critical implications for policy, practice, and research priorities. The analysis of quantitative findings highlights a significant link between climate variability, extreme weather events, and rising food insecurity levels. Studies have demonstrated that drought events lead to considerable reductions in agricultural output, directly contributing to increased food shortages at the household level (Deressa et al. 2011; Wheeler and von Braun 2013). Moreover, the growing frequency of floods has been shown to disrupt local market functions and supply chains, further complicating efforts to address food insecurity (Gebrehiwot and van der Veen 2013a, 2013b, 2013c).

Beyond Ethiopia, similar impacts have been reported in other climate‐vulnerable regions, including Sub‐Saharan Africa, South Asia, and parts of Latin America, where recurrent droughts, erratic rainfall, floods, and rising temperatures have reduced agricultural productivity, undermined rural livelihoods, and increased household food insecurity. This evidence shows that climate‐induced food system disruptions are a global issue affecting mainly rain‐fed agriculture, highlighting Ethiopia's experience within broader climate‐resilient food system challenges and the need to align national adaptation efforts with global strategies (IPCC 2022a, 2022b; FAO, IFAD, et al. 2023).

#### 3.8.1 Policy Implications

The review underscores the urgent need for climate adaptation policies that prioritize food security. Policymakers should integrate climate risk assessments into both national and regional development frameworks. One critical recommendation is the implementation of early warning systems to monitor and address the impacts of droughts and floods. These systems could significantly minimize the negative consequences on food availability and access, particularly in vulnerable regions (Conway and Schipper 2011). Additionally, the formulation of policies that promote sustainable land management and water conservation will be vital in mitigating the adverse effects of climate change on agricultural productivity, ensuring long‐term food security (Bryan et al. 2009).

Recent international evidence indicates that effective climate adaptation requires integrated food system approaches combining climate information services, risk‐informed agricultural planning, resilient infrastructure, social protection, and ecosystem‐based adaptation. Evidence from countries such as Kenya, Bangladesh, India, and Niger shows that coordinated investments in climate‐smart agriculture, weather‐index insurance, and climate‐informed extension services enhance smallholder adaptive capacity and reduce vulnerability (IPCC 2022a, 2022b; FAO et al. 2023). These experiences offer important lessons for strengthening Ethiopia's climate adaptation and food security policies in line with local socio‐economic and environmental conditions.

#### 3.8.2 Practical Implications

On a practical level, the review emphasizes the importance of community‐driven interventions. Strengthening the resilience of smallholder farmers is essential, and this can be achieved by improving access to climate‐resilient crop varieties and enhancing irrigation technologies. Evidence suggests that drought‐tolerant maize varieties have improved crop yields by up to 30% in areas prone to droughts, thus significantly boosting food security (Fisher et al. 2015). Moreover, expanding small‐scale irrigation systems has shown positive effects on household food security and income levels (Teshome et al. 2019). Alongside these interventions, capacity‐building programs aimed at enhancing farmers' knowledge of climate‐smart agricultural practices are necessary to foster long‐term resilience.

International evidence shows that integrating improved seed varieties with conservation agriculture, agroforestry, diversified cropping systems, efficient water management, and digital climate advisory services significantly enhances farm resilience to climate variability. In African and Asian contexts, such integrated approaches have increased productivity, improved livelihood diversification, and reduced household vulnerability to climate shocks (Lipper et al. 2014; IPCC 2022a, 2022b). Scaling up these evidence‐based interventions in Ethiopia, adapted to local agroecological conditions, could further strengthen food system resilience and support sustainable rural development.

#### 3.8.3 Research Priorities

The review also identifies several important research gaps that need to be addressed in future studies. First, there is a need for longitudinal research that examines the long‐term effects of climate variability on food security. Second, more research is required on the intersection of gender, climate risk, and food insecurity, in order to develop tailored interventions for vulnerable populations (Smith et al. 2014). At last, studies exploring the effectiveness of different adaptation strategies at the local level are essential for creating scalable solutions that can be applied across diverse regions (Thornton et al. 2014).

Future research should emphasize comparative cross‐country analyses to assess how governance systems, agricultural policies, institutional capacities, and adaptation investments shape food system resilience under climate change. Linking evidence from Ethiopia with other climate‐vulnerable regions would enhance generalizability and help identify transferable best practices. In addition, interdisciplinary approaches integrating climate science, agricultural economics, nutrition, remote sensing, and socio‐economic vulnerability analysis are needed to better capture the complex interactions between climate change, food systems, and rural livelihoods. Expanded use of geospatial analysis, climate modeling, and scenario‐based approaches would further strengthen evidence‐based policy and adaptation planning (IPCC 2022a, 2022b; FAO et al. 2023).

### 3.9 Limitations of the Systematic Review

Despite providing a comprehensive synthesis of the available evidence on the impacts of climate change on Ethiopia's food systems, this systematic review has several limitations that should be considered when interpreting the findings. First, although the review was conducted and reported in accordance with the Preferred Reporting Items for Systematic Reviews and Meta‐Analyses (PRISMA 2020) guidelines to ensure methodological transparency and reproducibility, the literature search was restricted to peer‐reviewed articles published in English and indexed in the selected electronic databases. Consequently, potentially relevant studies published in other languages or available as gray literature (e.g., government reports, technical reports, conference proceedings, and institutional publications) may not have been captured, introducing the possibility of language and publication bias (Page et al. 2021).

Second, considerable heterogeneity was observed among the included studies with respect to research design, geographical coverage, climatic variables, food system components, vulnerability indicators, analytical methods, and reported outcome measures. This methodological and contextual diversity precluded statistical pooling of the findings through meta‐analysis. Therefore, the evidence was synthesized using the Synthesis Without Meta‐analysis (SWiM) reporting guideline, which facilitates transparent narrative synthesis but does not provide pooled effect estimates, confidence intervals, or formal assessments of statistical heterogeneity (Campbell et al. 2020).

Third, the reviewed studies covered diverse agroecological zones, socioeconomic settings, and agricultural production systems across Ethiopia. Such regional heterogeneity may influence the magnitude and direction of reported climate change impacts and adaptation responses, limiting the generalizability of the findings to all regions and production systems within the country. Differences in data sources, climate scenarios, study periods, and methodological approaches may also have contributed to variations in reported outcomes across studies.

Fourth, although methodological quality assessment was conducted using predefined criteria to ensure the inclusion of reliable evidence, the conclusions of this review remain dependent on the quality, completeness, and reporting standards of the available primary studies. Variations in study quality, reporting practices, and potential selective publication of statistically significant findings may have influenced the overall evidence synthesis. As with most systematic reviews and based on published literature, the possibility of publication bias cannot be completely excluded (Higgins et al. 2022).

Finally, this review synthesized evidence published up to the final search date and therefore may not include recently published studies that could further refine current understanding of climate change impacts, vulnerability patterns, and adaptation strategies in Ethiopia's food systems. Future systematic reviews incorporating newly emerging evidence and, where sufficient homogeneous data are available, quantitative meta‐analyses would provide more robust estimates of climate change impacts and strengthen the evidence base for policy and practice.

## 4 Conclusion and Recommendations

This review highlights the complex relationship between climate risks and food insecurity in Ethiopia, synthesizing a range of quantitative evidence that emphasizes the urgency of addressing these issues. The key findings suggest that climate risks, including erratic rainfall, prolonged droughts, and flooding, have a severe impact on agricultural productivity, thereby exacerbating food insecurity, particularly for rural households that depend on rain‐fed agriculture. Multiple studies consistently demonstrate a strong relationship between climate variability and reduced crop yields, lower household income, and overall food shortages (Abebe et al. 2020; Tesfaye and Gebreselassie 2021). Although these findings provide strong evidence, the synthesis is constrained by variation in study coverage, temporal differences, and uneven spatial representation across agro‐ecological zones of Ethiopia.

Furthermore, the review points to increasing vulnerability among marginalized groups, where limited adaptive capacity is exacerbated by socio‐economic factors such as poor access to climate‐resilient agricultural technologies, insufficient infrastructure, and lack of policy support. Evidence strongly suggests that without significant intervention, food insecurity in Ethiopia is likely to worsen as climate risks become more intense (World Bank 2022a, 2022b, 2022c, 2022d). The review stresses the critical need for immediate action to address the impacts of climate risks on food security. Ethiopia is at a pivotal moment, where proactive and comprehensive measures are necessary to mitigate these risks and protect livelihoods. The findings emphasize the importance of investing in climate‐smart agricultural practices, establishing early warning systems, and improving water resource management to build resilience at the community and household levels.

In addition, stronger integration of ecosystem‐based adaptation and landscape restoration approaches is essential to enhance long‐term ecosystem resilience and sustain agricultural productivity under increasing climate stress.

A multi‐sectoral approach is crucial in tackling food insecurity in the face of climate risks. Successful mitigation of food insecurity requires coordinated efforts from government bodies, non‐governmental organizations, international development partners, and local communities. Policies should focus on integrated approaches that combine agricultural development with climate adaptation strategies. Expanding access to microfinance for smallholder farmers and fostering public–private partnerships can play a vital role in enhancing the resilience of vulnerable populations (Gebrekidan et al. 2023). Future policy frameworks should further prioritize institutionalizing climate adaptation planning within national and regional agricultural strategies, ensuring that climate information services, risk financing mechanisms, and climate‐smart extension systems are systematically scaled up.

In conclusion, addressing climate risks and food insecurity in Ethiopia demands urgent and comprehensive action. Through fostering collaboration across sectors and utilizing evidence‐based strategies, Ethiopia can pave the way for a more resilient and food‐secure future.

Future research should focus on harmonized longitudinal datasets, high‐resolution spatial analysis of vulnerability hotspots, and improved integration of socio‐economic and biophysical models to better capture localized climate–food security dynamics.

## Funding

The authors have nothing to report.

## Conflicts of Interest

The authors declare no conflicts of interest.

## Data Availability

No new primary data were generated during this study. The data supporting the findings of this systematic review and meta‐analysis were extracted from previously published peer‐reviewed studies and publicly available sources cited throughout the manuscript. The original source data are available from the cited publications.
